# tRF-AspGTC Promotes Intracranial Aneurysm Formation by Controlling TRIM29-Mediated Galectin-3 Ubiquitination

**DOI:** 10.34133/research.0574

**Published:** 2025-01-07

**Authors:** Chao Wang, Bing Yu, Han Zhou, Huanting Li, Shifang Li, Xiaolu Li, Wentao Wang, Yugong Feng, Tao Yu

**Affiliations:** ^1^ Department of Neurosurgery and Institute for Translational Medicine, The Affiliated Hospital of Qingdao University, Qingdao 266000, People’s Republic of China.; ^2^; Department of Ophthalmology, The Affiliated Hospital of Qingdao University, Qingdao 266000, People’s Republic of China.; ^3^ Department of Critical Care Medicine, Shandong Provincial Hospital Affiliated to Shandong First Medical University, Jinan 250021, People’s Republic of China. ^4^Department of Cardiac Ultrasound, The Affiliated Hospital of Qingdao University, Qingdao 266000, People’s Republic of China.

## Abstract

Transfer RNA-derived small RNAs, a recently identified class of small noncoding RNAs, play a crucial role in regulating gene expression and are implicated in cerebrovascular diseases. However, the specific biological roles and mechanisms of transfer RNA-derived small RNAs in intracranial aneurysms (IAs) remain unclear. In this study, we identified that the transfer RNA-Asp-GTC derived fragment (tRF-AspGTC) is highly expressed in the IA tissues of both humans and mice. tRF-AspGTC promotes IA formation by facilitating the phenotypic switching of vascular smooth muscle cells, increasing of matrix metalloproteinase 9 expression, and inducing of oxidative stress and inflammatory responses. Mechanistically, tRF-AspGTC binds to galectin-3, inhibiting tripartite motif 29-mediated ubiquitination and stabilizing galectin-3. This stabilization activates the toll-like receptor 4/MyD88/nuclear factor kappa B pathway, further driving phenotypic switching and inflammation. Clinically, circulating exosomal tRF-AspGTC demonstrates strong diagnostic efficacy for IAs and is identified as an independent risk factor for IA occurrence. These findings highlight the potential of tRF-AspGTC as a promising diagnostic biomarker and therapeutic target for IAs.

## Introduction

Intracranial aneurysms (IAs) are characterized by the abnormal dilation of the cerebral artery wall and represent a common cerebrovascular disorder, with an estimated incidence of approximately 3.2% in the general population [1–3]. When an IA ruptures, it can result in subarachnoid hemorrhage (SAH), a life-threatening event associated with a mortality rate of up to 40% and a disability rate as high as 50%, posing a marked public health challenge [4,5]. Pathologically, IAs are associated with several key factors, including hemodynamic stress, inflammation, vascular smooth muscle cell (VSMC) phenotypic switching, and extracellular matrix (ECM) degradation [6,7]. One of the critical pathogenic mechanisms involves the generation of reactive oxygen species (ROS), which contribute to VSMC dysfunction, endothelial cell injury, and inflammatory responses [8,9]. The clinical management of IAs primarily relies on surgical interventions, such as craniotomy clipping and endovascular procedures [10,11]. Although these surgical methods are effective, they carry considerable risks. Craniotomy clipping is a highly invasive procedure that requires a lengthy recovery period and may be accompanied by complications such as infection and nerve damage [12,13]. Endovascular procedures, while less invasive, still pose risks of rebleeding and embolization failure [14,15]. Furthermore, these surgical approaches may not be suitable for elderly patients or individuals with severe comorbidities [16]. Additionally, the diagnosis of IAs primarily depends on invasive vascular imaging techniques, such as computed tomography angiography or digital subtraction angiography, which lack effective clinical screening indicators and are associated with high costs and risks related to radiation exposure and contrast agents [17]. Given the absence of reliable clinical screening markers and pharmacological therapies, it is imperative to identify novel biomarkers for IA diagnosis and to explore valuable therapeutic targets.

Transfer RNA-derived small RNAs (tsRNAs), a newly identified class of small noncoding RNAs, originate from ribonuclease-mediated cleavage of precursor or mature transfer RNAs (tRNAs) [18,19]. tsRNAs have several natural advantages, including high stability and abundant expression levels. They are less susceptible to degradation by RNases and exhibit high expression with strong specificity under certain physiological or pathological conditions [20]. These characteristics make them promising candidates for regulating various biological processes and serving as potential biomarkers [21]. tsRNAs perform a range of biological functions by interacting with messenger RNAs (mRNAs) or proteins, thereby influencing gene expression, translation, and epigenetic modifications [22]. Notably, tsRNAs have been shown to play important roles in the progression of diseases, including cancer and cardiovascular disorders, as evidenced by previous studies [23–27]. For instance, AS-tDR-007333 promotes lung cancer malignancy through the HSPB1–MED29 and ELK4–MED29 pathways [24]. Additionally, 5′-tiRNA-Cys-GCA regulates VSMC proliferation and phenotypic switching by targeting STAT4 in aortic dissection [25]. However, the role of tsRNAs in IAs remains unexplored.

Galectin-3, encoded by the lectin galactoside-binding soluble 3 (LGALS3) gene, is a β-galactoside-binding protein [28]. It is the sole chimeric member of the galectin family, characterized by a single C-terminal carbohydrate recognition domain and a nonlectin collagen-like N-terminal region [29,30]. Galectin-3 interacts with various glycosylated receptors, triggering diverse signaling pathways that include immune cell activation, cytokine secretion, cell migration and proliferation, and apoptosis [31]. Research has indicated that galectin-3 can stimulate macrophage activation, elastin degradation, and smooth muscle cell apoptosis, thereby facilitating the development of abdominal aortic aneurysms [32,33]. Moreover, elevated galectin-3 expression has been observed in patients with SAH caused by IA rupture [34]. This increased expression is associated with exacerbated blood–brain barrier disruption, highlighting a strong correlation between galectin-3 and the progression of IAs, as well as poor prognostic outcomes [35].

In this study, we identified a direct correlation between the transfer RNA-Asp-GTC derived fragment (tRF-AspGTC) and the pathogenesis of IAs. tRF-AspGTC interacts with galectin-3, inhibiting tripartite motif 29 (TRIM29)-induced degradation of galectin-3, thereby leading to an up-regulation of its expression. This interaction promotes galectin-3-dependent toll-like receptor 4 (TLR4) activation, subsequently initiating the TLR4/MyD88/nuclear factor kappa B (NF-κB) pathway. As a result, this pathway induces VSMC phenotypic switching, matrix metalloproteinase (MMP) activation, oxidative stress, and inflammation. Additionally, the detection of tRF-AspGTC in plasma exosomes from IA patients highlights its potential as a promising biomarker for IA diagnosis. These findings provide essential insights into the role of the tRF-AspGTC/galectin-3/TLR4/NF-κB axis in IA formation.

## Results

### tRF-AspGTC is up-regulated in IAs

**Fig. 1.**
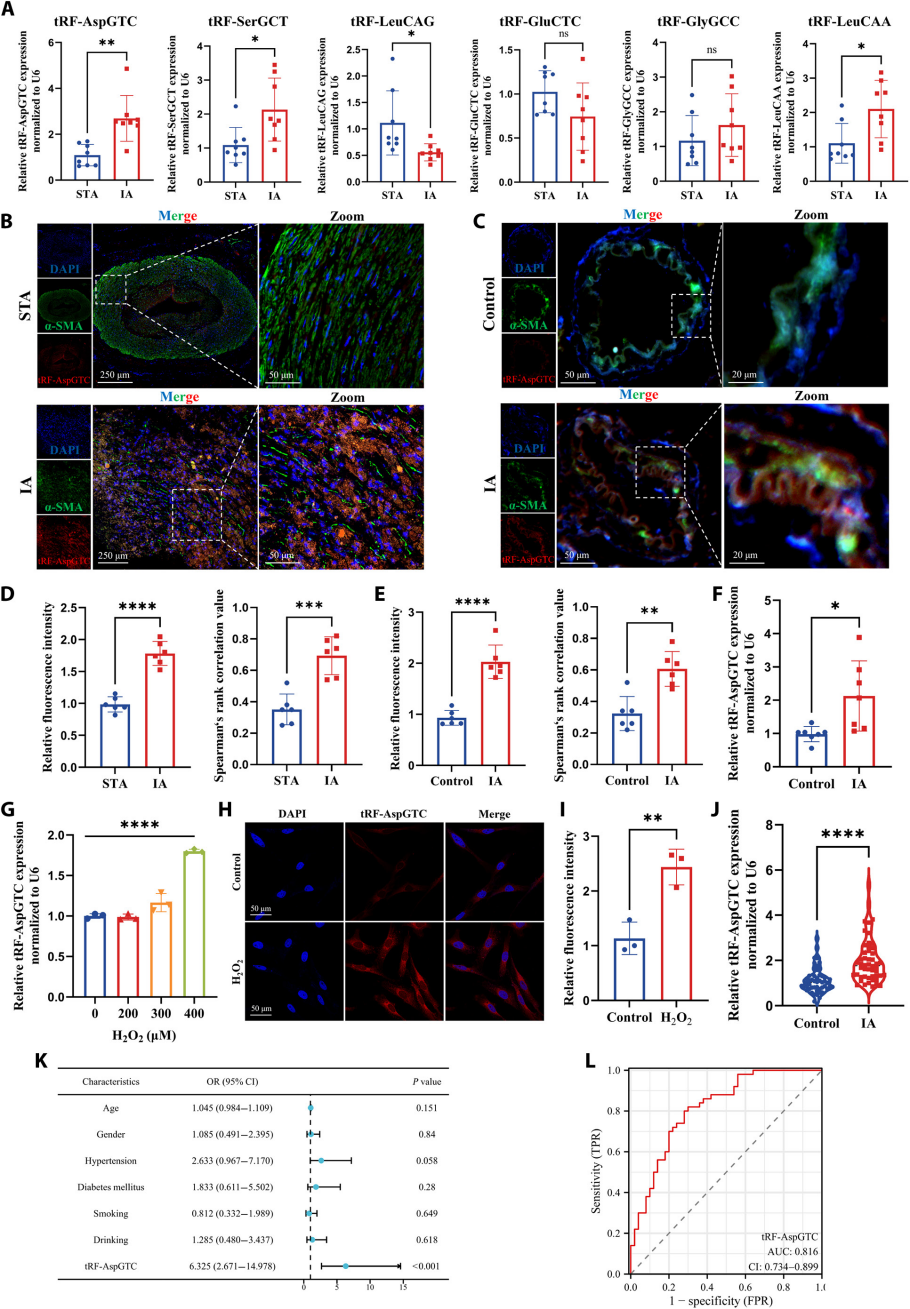
The transfer RNA-Asp-GTC derived fragment (tRF-AspGTC) is up-regulated in intracranial aneurysms (IAs). (A) Reverse transcription quantitative polymerase chain reaction (RT-qPCR) analysis of transfer RNA-derived small RNA (tsRNA) expression in human superficial temporal artery (STA) and IA tissues; *n* = 8 per group. (B) Fluorescence in situ hybridization (FISH) and immunofluorescence (IF) detection of tRF-AspGTC (red) and alpha smooth muscle actin (α-SMA) (green) colocalization, showing differential expression of tRF-AspGTC in human STA and IA tissues (scale bars = 250 and 50 μm). (C) FISH and IF detection of tRF-AspGTC (red) and α-SMA (green) colocalization, highlighting the expression differences of tRF-AspGTC in the brain arteries of normal and IA mice (scale bars = 50 and 20 μm). (D and E) Quantitative analysis of relative fluorescence intensity and Pearson correlation analysis of (B) and (C); *n* = 6 per group. (F) RT-qPCR analysis of tRF-AspGTC expression levels in the brain arteries of normal and IA mice; *n* = 7 per group. (G) RT-qPCR analysis of tRF-AspGTC expression levels in vascular smooth muscle cells (VSMCs) treated with varying concentrations of hydrogen peroxide; *n* = 3 per group. (H) FISH detection of tRF-AspGTC expression in VSMCs post 400 μM hydrogen peroxide treatment (scale bar = 50 μm). (I) Quantitative analysis of the relative fluorescence intensity of (H); *n* = 6 per group. (J) RT-qPCR analysis of tRF-AspGTC expression in plasma exosomes from healthy individuals and IA patients; *n* = 50 per group. (K) Multivariate logistic regression analysis of plasma exosomal tRF-AspGTC expression levels in IA patients. (L) Receiver operating characteristic (ROC) curve of plasma exosomal tRF-AspGTC expression in IA patients. *P < 0.05; **P < 0.01; ***P < 0.001; ****P < 0.0001. ns, not significant; DAPI, 4′,6-diamidino-2-phenylindole; OR, odds ratio; CI, confidence interval; TPR, true-positive rate; FPR, false-positive rate; AUC, area under the curve.

To identify tsRNAs influencing IA formation, plasma exosomes were collected and 4 pairs of IA patients and healthy volunteers were randomly selected for high-throughput sequencing. Six differentially expressed tsRNAs were subsequently validated using reverse transcription quantitative polymerase chain reaction (RT-qPCR) (Fig. 1A) in IA and superficial temporal artery (STA) tissues. Among these, tRF-AspGTC exhibited the highest level of differential expression between the 2 groups. It is a 35-nt-long tRNA-derived fragment derived from the cleavage of tRNA-Asp-GTC (Fig. S1). Fluorescence in situ hybridization (FISH) analysis revealed elevated tRF-AspGTC expression in both human (Fig. 1B and D) and mouse IA tissues (Fig. 1C and E), with colocalization analysis confirming its predominant localization in VSMCs. Additionally, tRF-AspGTC demonstrated up-regulation in the brain vascular tissues of IA mice induced by elastase (Fig. 1F). In response to oxidative stress, VSMCs transition from a contractile to a synthetic phenotype, activating MMPs and inflammatory responses [36]. Treatment of VSMCs with a gradient of hydrogen peroxide concentration for 24 h resulted in decreased expression of contractile marker proteins (myosin heavy chain [MHC], alpha smooth muscle actin [α-SMA], and calponin 1 [CNN1]) (Fig. S2A and B) and increased expression of matrix metalloproteinase 2 (MMP2) and matrix metalloproteinase 9 (MMP9) (Fig. S2A and C). Notably, we assessed the RNA levels of hydrogen peroxide-treated VSMCs and observed an increase in tRF-AspGTC expression at a concentration of 400 μM (Fig. 1G). FISH analysis (Fig. 1H and I) of VSMCs corroborated these findings, revealing predominant cytoplasmic localization of tRF-AspGTC. Similarly, angiotensin II treatment also induced increased tRF-AspGTC expression in VSMCs (Fig. S2D). The increased expression of tRF-AspGTC in IA suggests its potential involvement in IA pathogenesis.

Plasma exosome tsRNAs have emerged as promising biomarkers due to their stability and high sensitivity [37–39]. RT-qPCR analysis of an independent cohort of IA patients (Fig. 1J) demonstrated elevated levels of tRF-AspGTC in their plasma exosomes compared to those of a matched healthy control group. Multivariate logistic regression analysis (Fig. 1K and Table S1) identified tRF-AspGTC as an independent risk factor for IA occurrence. Furthermore, receiver operating characteristic (ROC) curve analysis (Fig. 1L) yielded an area under the curve of 0.816, underscoring the significant potential of plasma exosome tRF-AspGTC as a reliable clinical diagnostic marker for IA.

### The role of tRF-AspGTC in regulating VSMC phenotypic switching, MMP expression, oxidative stress, and inflammation

**Fig. 2.**
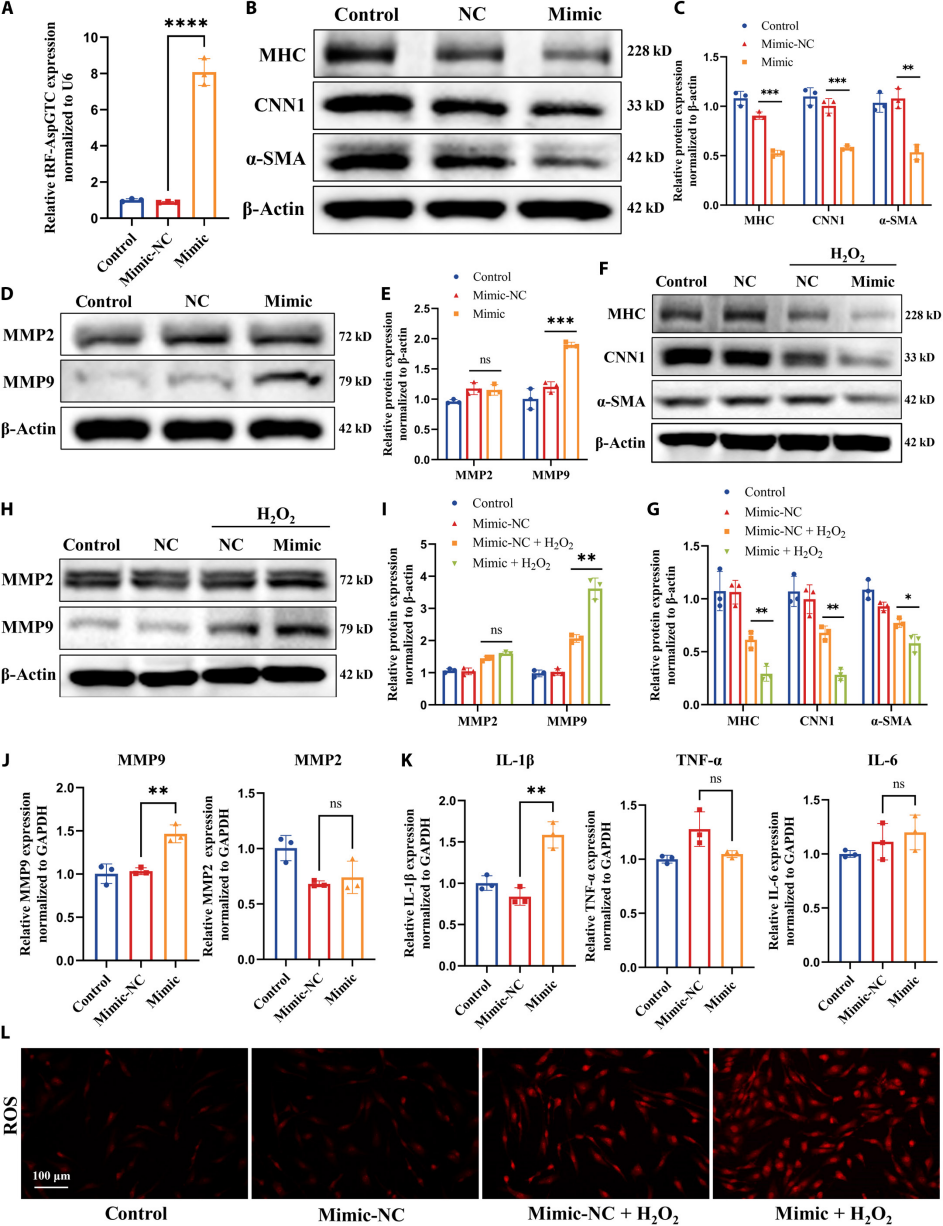
tRF-AspGTC overexpression induces VSMC phenotypic switching and inflammation. (A) RT-qPCR analysis of overexpression efficiency following transfection with a tRF-AspGTC mimic; *n* = 3 per group. (B) Western blot (WB) analysis of contractile VSMC marker proteins myosin heavy chain (MHC), calponin 1 (CNN1), and α-SMA expression levels after tRF-AspGTC overexpression. (C) Quantitative analysis of (B); *n* = 3 per group. (D) WB analysis of matrix metalloproteinase 2 (MMP2) and matrix metalloproteinase 9 (MMP9) protein expression levels. (E) Quantitative analysis of (D); *n* = 3 per group. (F) WB analysis of MHC, CNN1, and α-SMA expression levels in VSMCs treated with 400 μM hydrogen peroxide for 24 h after tRF-AspGTC overexpression. (G) Quantitative analysis of (F); *n* = 3 per group. (H) WB analysis of MMP2 and MMP9 protein expression levels. (I) Quantitative analysis of (H); *n* = 3 per group. (J) RT-qPCR analysis of MMP2 and MMP9 messenger RNA (mRNA) levels following tRF-AspGTC overexpression; *n* = 3 per group. (K) RT-qPCR analysis of inflammatory cytokines interleukin 1 beta (IL-1β), tumor necrosis factor alpha (TNF-α), and interleukin 6 (IL-6) mRNA expression levels; *n* = 3 per group. (L) Measurement of reactive oxygen species (ROS) generation in VSMCs treated with hydrogen peroxide following tRF-AspGTC overexpression. *P < 0.05; **P < 0.01; ***P < 0.001; ****P < 0.0001. NC, negative control; GAPDH, glyceraldehyde-3-phosphate dehydrogenase.

To investigate the biological functions of tRF-AspGTC in VSMCs, we conducted both gain-of-function and loss-of-function experiments. For the gain-of-function analysis, tRF-AspGTC mimics were synthesized and transfected into VSMCs, leading to a significant increase in tRF-AspGTC expression (Fig. 2A). Under physiological conditions, tRF-AspGTC overexpression resulted in a marked reduction of contractile markers (MHC, α-SMA, and CNN1) in VSMCs (Fig. 2B and C), accompanied by elevated MMP9 expression. In contrast, MMP2 levels remained unchanged (Fig. 2D and E). At the RNA level, a similar pattern was observed, with increased MMP9 mRNA expression and no significant changes in MMP2 mRNA levels (Fig. 2J). Furthermore, tRF-AspGTC overexpression significantly up-regulated the inflammatory cytokine interleukin 1 beta (IL-1β), while the expression of tumor necrosis factor alpha (TNF-α) and interleukin 6 (IL-6) remained unaffected (Fig. 2K). Under pathological conditions, tRF-AspGTC overexpression markedly exacerbated hydrogen peroxide-induced phenotypic switching of VSMCs (Fig. 2F and G), enhanced IL-1β expression (Fig. S3A), increased MMP9 expression at both the protein and RNA levels (Fig. 2H and I and Fig. S3B), and elevated ROS production (Fig. 2L).

**Fig. 3.**
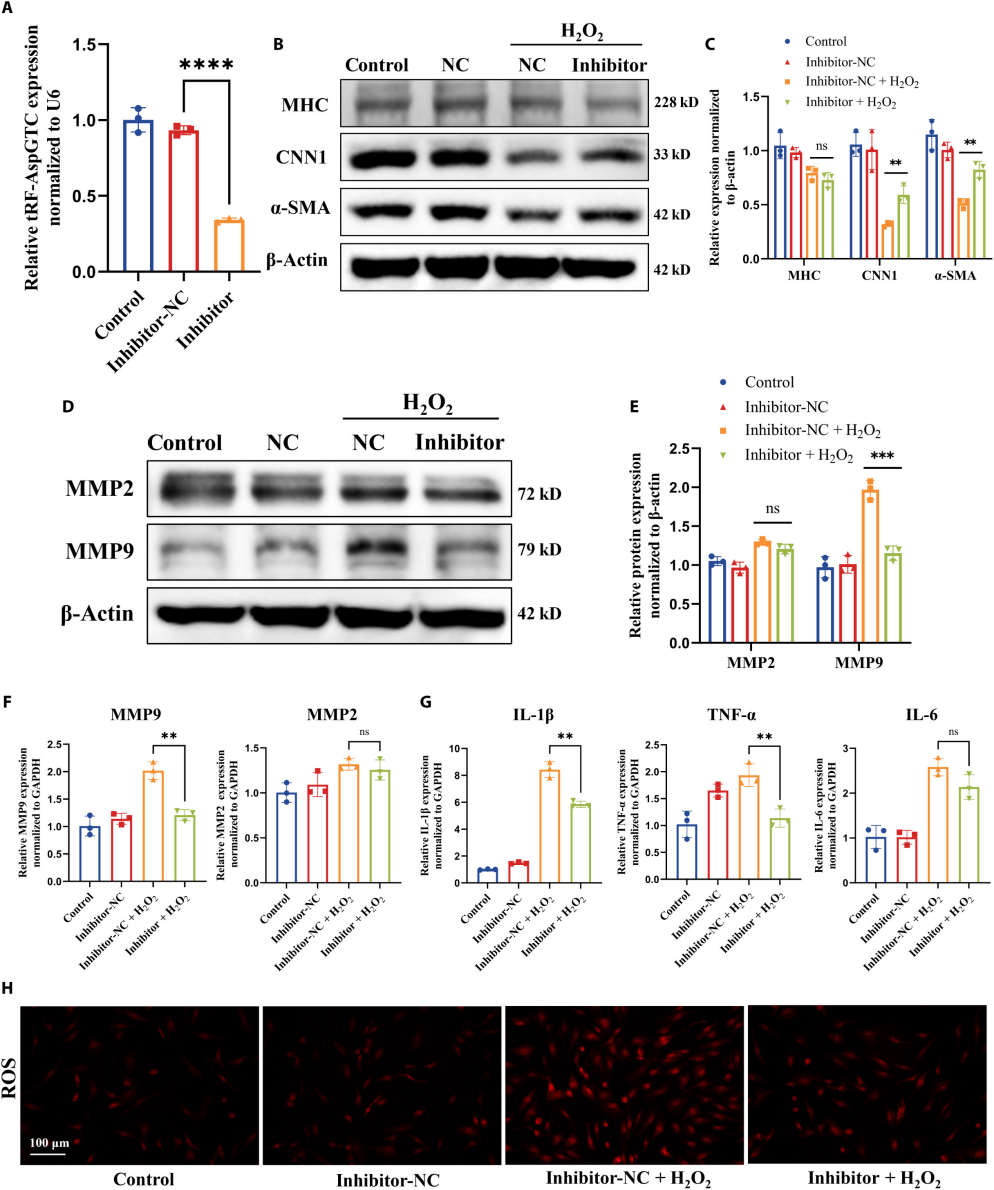
Knocking down tRF-AspGTC alleviates hydrogen peroxide-induced VSMC phenotypic switching, oxidative stress, and inflammation. (A) Knockdown efficiency of tRF-AspGTC inhibitor transfection detected by RT-qPCR; *n* = 3 per group. (B) WB analysis of the expression levels of contractile VSMC marker proteins MHC, CNN1, and α-SMA in VSMCs treated with 400 μM hydrogen peroxide for 24 h following tRF-AspGTC knockdown. (C) Quantitative analysis of (B); *n* = 3 per group. (D) WB analysis of MMP2 and MMP9 protein expression levels. (E) Quantitative analysis of (D); *n* = 3 per group. (F) RT-qPCR analysis of MMP2 and MMP9 mRNA levels; *n* = 3 per group. (G) RT-qPCR analysis of the mRNA levels of inflammatory cytokines IL-1β, TNF-α, and IL-6; *n* = 3 per group. (H) Detection of ROS generation in VSMCs. **P < 0.01; ***P < 0.001; ****P < 0.0001.

For the loss-of-function analysis, inhibitors targeting tRF-AspGTC were constructed and transfected into VSMCs, resulting in significant down-regulation of tRF-AspGTC expression (Fig. 3A). Suppression of tRF-AspGTC expression effectively mitigated hydrogen peroxide-induced phenotypic switching in VSMCs (Fig. 3B and C). This intervention reversed the hydrogen peroxide-induced up-regulation of MMP9 expression at both the protein (Fig. 3D and E) and mRNA levels (Fig. 3F) while simultaneously reducing the expression of IL-1β and TNF-α (Fig. 3G). Additionally, tRF-AspGTC knockdown significantly attenuated hydrogen peroxide-induced ROS production (Fig. 3H). In conclusion, these findings underscore the pivotal role of tRF-AspGTC in regulating VSMC phenotypic switching, MMP9 expression, and oxidative stress and inflammatory responses. This suggests that tRF-AspGTC may contribute to the formation of IA by modulating VSMC function.

### tRF-AspGTC interacts with galectin-3 in VSMCs

**Fig. 4.**
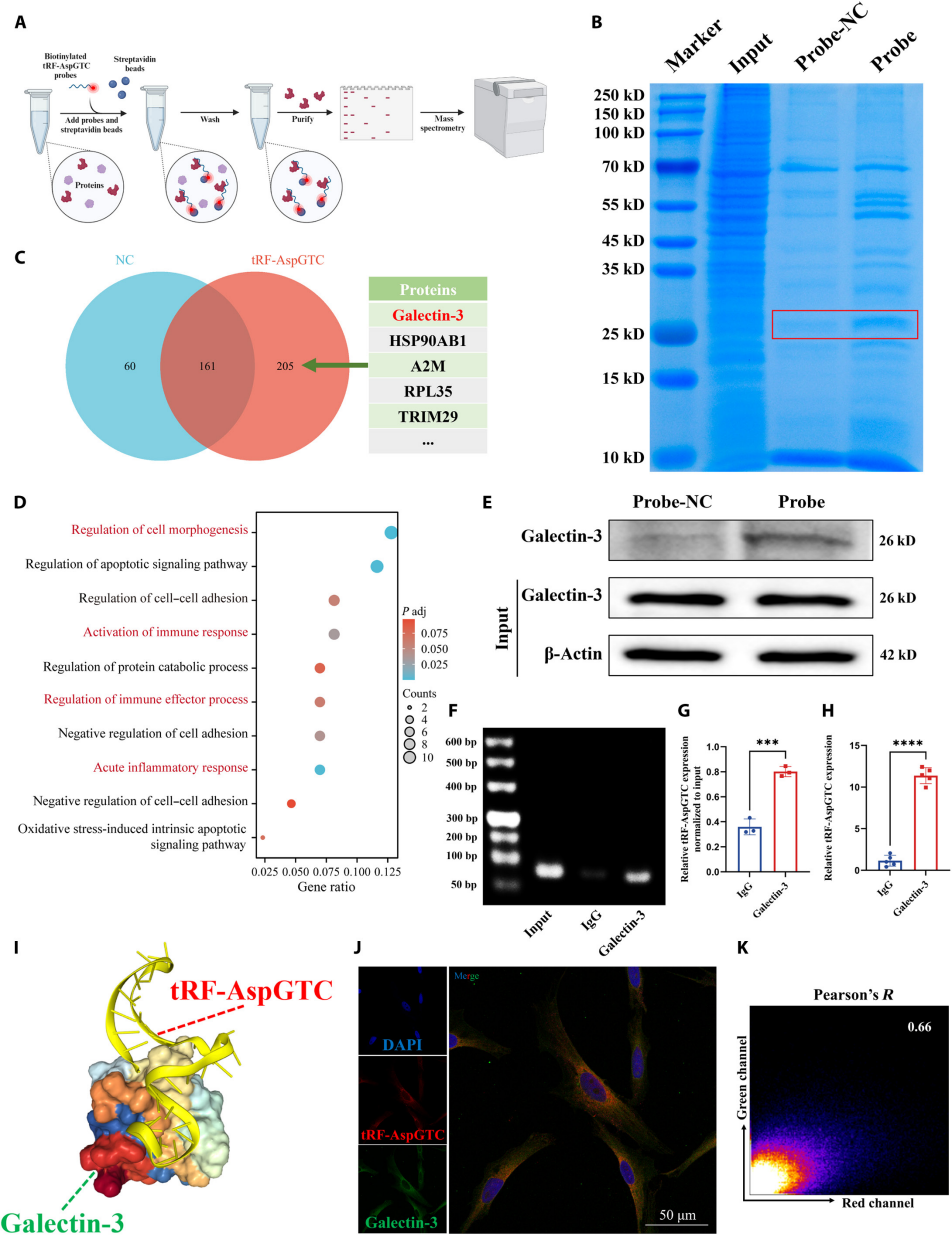
tRF-AspGTC specifically binds to galectin-3 in VSMCs. (A) Schematic diagram of the RNA pulldown and protein mass spectrometry analysis workflow. (B) Proteins specifically bound to tRF-AspGTC were separated using sodium dodecyl sulfate polyacrylamide gel electrophoresis (SDS-PAGE), with significantly different protein bands highlighted in red boxes. (C) Venn diagram showing the mass spectrum results and a table listing RNA-binding proteins with high binding scores. (D) Functional enrichment analysis of tRF-AspGTC-specific binding proteins. (E) Screening of tRF-AspGTC-specific binding proteins via RNA pulldown followed by WB detection of galectin-3. (F) Detection of tRF-AspGTC using polymerase chain reaction (PCR) after RNA immunoprecipitation. (G) Quantitative analysis of (F); *n* = 3 per group. (H) Detection of tRF-AspGTC using RT-qPCR after RNA immunoprecipitation; *n* = 5 per group. (I) Predicted 3-dimensional (3D) structure of the tRF-AspGTC–galectin-3 complex. (J) Colocalization detection of tRF-AspGTC (red) and galectin-3 (green) via FISH and IF. (K) Pearson correlation analysis of the fluorescence colocalization, with a correlation coefficient of 0.66. ***P < 0.001; ****P < 0.0001. TRIM29, tripartite motif 29; IgG, immunoglobulin G.

Initially, tsRNAs were thought to function similarly to microRNAs, primarily binding to Ago family proteins and targeting mRNAs with complementary sequences to exert posttranscriptional silencing [40–42]. Recent research has revealed that tsRNAs can also interact directly with RNA-binding proteins (RBPs) to perform biological functions [24]. To elucidate the mechanisms through which tRF-AspGTC exerts its biological functions in VSMCs, we performed RNA pulldown experiments using biotinylated tRF-AspGTC probes, followed by mass spectrometry analysis (Fig. 4A). Our results identified 205 RBPs precipitated by tRF-AspGTC, including galectin-3, HSP90AB1, A2M, RPL35, and TRIM29 (Fig. 4B and C). Functional enrichment analysis (Fig. 4D) of tRF-AspGTC-specific RBPs revealed their association with pathways such as “regulation of cell morphogenesis” and “activation of immune response”. We specifically focused on galectin-3 due to its high matching score and its well-documented role in the inflammatory response, which is closely associated with IA formation and rupture [35,43]. Western blot (WB) analysis (Fig. 4E) following RNA pulldown confirmed the specific binding between tRF-AspGTC and galectin-3. Furthermore, RNA immunoprecipitation–polymerase chain reaction (Fig. 4F and G) and RNA immunoprecipitation–qPCR (Fig. 4H) using galectin-3 antibodies further validated this interaction. Protein–RNA docking analysis (http://hdock.phys.hust.edu.cn/) provided additional evidence of a direct interaction between tRF-AspGTC and galectin-3 (Fig. 4I). To further delineate their spatial correlation, fluorescence colocalization studies were conducted. These studies revealed the colocalization of red fluorescence from tRF-AspGTC with green fluorescence from galectin-3 in the cytoplasm, indicating a direct spatial interaction (Fig. 4J and K). These findings suggest that tRF-AspGTC may exert its biological functions by directly binding to galectin-3 in VSMCs.

### tRF-AspGTC stabilizes galectin-3 by preventing TRIM29-mediated ubiquitination degradation in VSMCs

**Fig. 5.**
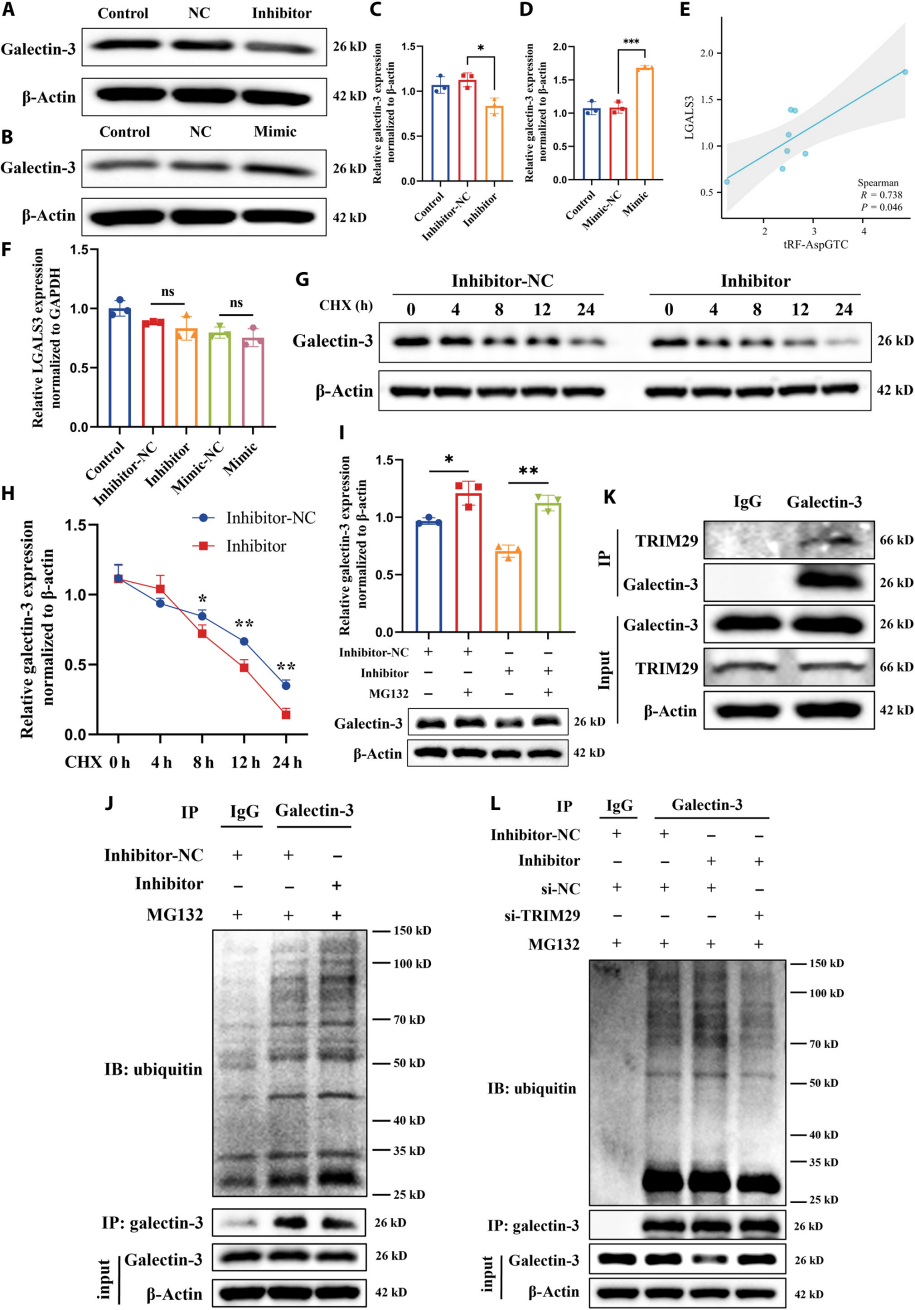
tRF-AspGTC stabilizes galectin-3 by preventing TRIM29-mediated ubiquitination degradation in VSMCs. (A) WB analysis of galectin-3 expression after tRF-AspGTC knockdown. (B) WB analysis of galectin-3 expression after tRF-AspGTC overexpression. (C and D) Quantitative analysis of (A) and (B); *n* = 3 per group. (E) Spearman correlation analysis of tRF-AspGTC and LGALS3 mRNA levels in human IA tissues; *n* = 8 per group. (F) RT-qPCR analysis of LGALS3 mRNA levels in VSMCs after tRF-AspGTC knockdown or overexpression; *n* = 3 per group. (G) WB analysis of galectin-3 expression in VSMCs transfected with inhibitor-NC or the inhibitor and treated with cycloheximide (CHX) (200 μM). (H) Quantitative analysis of (G); *n* = 3 per group. (I) WB analysis of galectin-3 expression in VSMCs transfected with or without tRF-AspGTC inhibitor following proteasome inhibition with MG132 treatment (20 μM) for 3 h; *n* = 3 per group. (J) WB analysis of galectin-3 expression and ubiquitination levels in VSMCs transfected with either inhibitor-NC or tRF-AspGTC inhibitor, followed by immunoprecipitation with a galectin-3 antibody. (K) WB analysis of TRIM29 immunoprecipitated by galectin-3. (L) WB analysis of galectin-3 expression and ubiquitination levels in cells transfected with inhibitor-NC, the inhibitor, si-NC, or si-TRIM29, followed by immunoprecipitation with a galectin-3 antibody. *P < 0.05; **P < 0.01; ***P < 0.001. si-NC, small interfering RNA negative control; LGALS3, lectin galactoside-binding soluble 3; si-TRIM29, small interfering RNA targeting tripartite motif containing 29; IP, immunoprecipitation; IB, immunoblotting.

After confirming the specific binding of tRF-AspGTC with galectin-3 in VSMCs, we sought to further elucidate the mechanisms underlying their interaction. WB revealed a decrease in galectin-3 protein levels following tRF-AspGTC knockdown (Fig. 5A and C) and an increase following tRF-AspGTC overexpression (Fig. 5B and D). Additionally, we assessed the RNA levels of tRF-AspGTC and galectin-3 in clinical IA tissues. Spearman correlation analysis (Fig. 5E) indicated a positive correlation between the expression of these 2 molecules. However, alterations in tRF-AspGTC expression through knockdown or overexpression did not affect galectin-3 mRNA levels (Fig. 5F), suggesting that tRF-AspGTC may not regulate galectin-3 expression via transcriptional pathways.

We explored the possibility of tRF-AspGTC exerting its effects through stabilizing galectin-3. To test this hypothesis, we employed a chase assay using cycloheximide (Fig. 5G and H), a protein translation inhibitor, and observed that galectin-3 protein stability decreased upon tRF-AspGTC silencing, indicating that tRF-AspGTC influences galectin-3 protein stability. MG132, a potent inhibitor of the ubiquitin–proteasome pathway, was used to investigate whether tRF-AspGTC affects galectin-3 stability through proteasomal or lysosomal pathways. The results showed that MG132 reversed the decrease in galectin-3 protein levels caused by tRF-AspGTC knockdown (Fig. 5I). These findings suggest that tRF-AspGTC stabilizes galectin-3 by preventing its proteasomal degradation. To investigate the possible influence of tRF-AspGTC on the ubiquitination and degradation of galectin-3, we performed immunoprecipitation assays on VSMCs with and without tRF-AspGTC silencing and evaluated ubiquitination levels. Our findings revealed that silencing tRF-AspGTC resulted in elevated levels of galectin-3 ubiquitination (Fig. 5J), indicating that tRF-AspGTC stabilizes galectin-3 by inhibiting its ubiquitination.

TRIM family proteins, functioning as E3 ubiquitin ligases, have shown a tendency to interact with galectins [43–45]. Previous research has reported an interaction between TRIM16 and galectin-3 in a UNC-51-like kinase 1-dependent manner [44]. Interestingly, in the mass spectrometry analysis of proteins specifically binding to tRF-AspGTC, TRIM29 was identified to interact with tRF-AspGTC. WB analysis following RNA pulldown confirmed their specific interaction (Fig. S4A). FISH and immunofluorescence (IF) further confirmed this interaction (Fig. S4B). Additionally, WB analysis showed that TRIM29 expression decreased following tRF-AspGTC overexpression (Fig. S4C and D). In addition, the immunoprecipitation results showed that galectin-3 can specifically bind to TRIM29 (Fig. 5K). We hypothesize that tRF-AspGTC may influence galectin-3 ubiquitination through TRIM29. To test this hypothesis, we constructed small interfering RNA (siRNA) targeting TRIM29 and validated its knockdown efficiency at both the mRNA (Fig. S4E) and protein levels (Fig. S4F and G). Subsequent dual-transfection experiments revealed that knocking down TRIM29 could reverse the decrease in galectin-3 protein levels caused by tRF-AspGTC knockdown (Fig. 5L). Additionally, we found that TRIM29 knockdown reduced the elevated ubiquitination levels of galectin-3 induced by tRF-AspGTC knockdown(Fig. 5L). In summary, we demonstrated that tRF-AspGTC can influence the expression of galectin-3 through TRIM29-mediated ubiquitination.

### tRF-AspGTC accelerates VSMC phenotypic switching, oxidative stress, and inflammation by targeting galectin-3

**Fig. 6.**
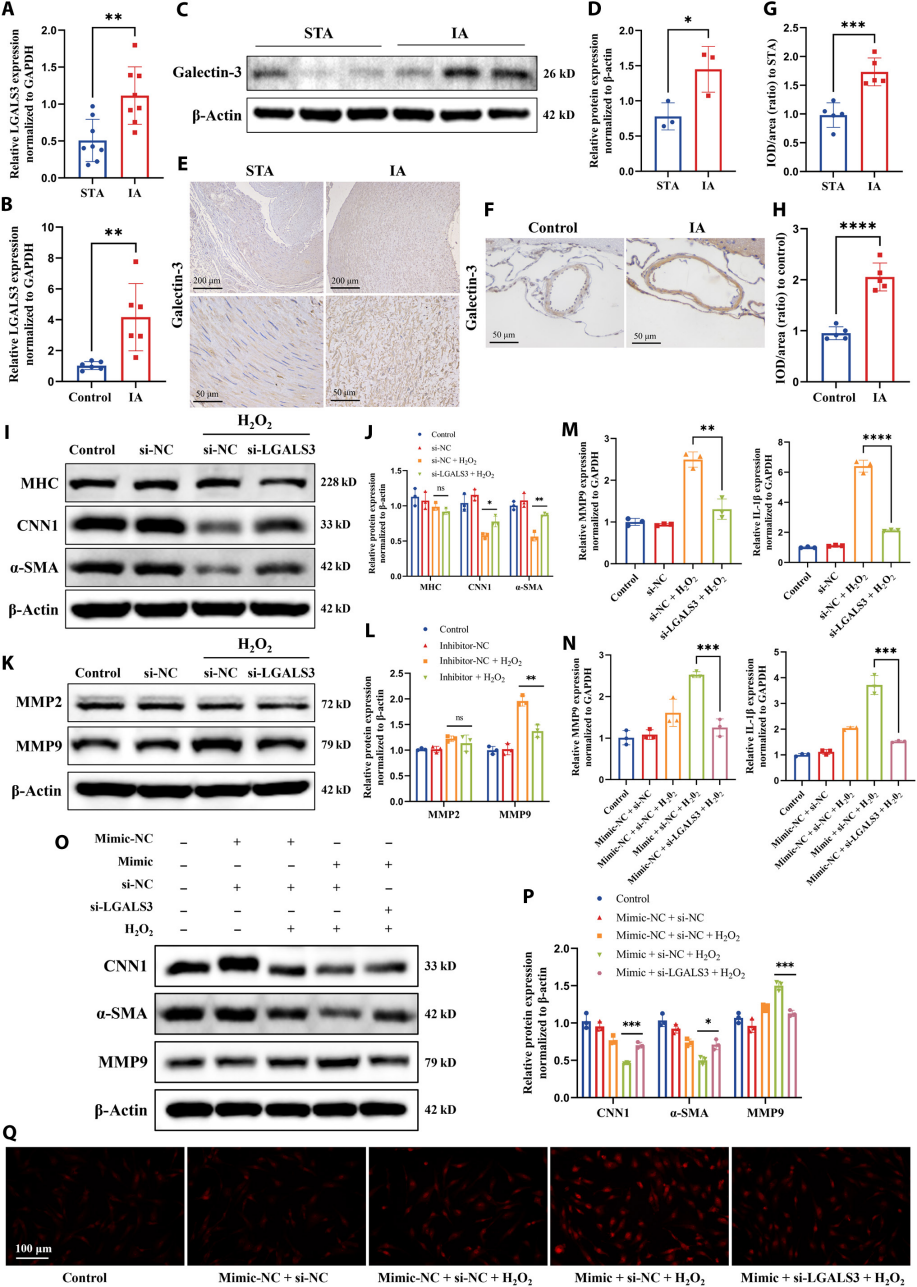
tRF-AspGTC accelerates VSMC phenotypic switching, oxidative stress, and inflammation by targeting galectin-3. (A) RT-qPCR analysis of LGALS3 mRNA levels in human STA and IA tissues; *n* = 8 per group. (B) RT-qPCR analysis of LGALS3 mRNA levels in the brain arteries of normal and IA mice; *n* = 6 per group. (C) WB analysis of galectin-3 expression levels in human STA and IA tissues. (D) Quantitative analysis of (C); *n* = 3 per group. (E) Immunohistochemical (IHC) detection of galectin-3 expression in human STA and IA tissues. (F) IHC detection of galectin-3 expression in the brain arteries of normal and IA mice. (G and H) Quantitative analysis of (E) and (F); *n* = 5 per group. (I) WB analysis of the expression levels of contractile VSMC marker proteins MHC, CNN1, and α-SMA in VSMCs treated with hydrogen peroxide following LGALS3 knockdown. (J) Quantitative analysis of (I); *n* = 3 per group. (K) WB analysis of MMP2 and MMP9 expression levels. (L) Quantitative analysis of (K); *n* = 3 per group. (M) RT-qPCR analysis of MMP9 and IL-1β mRNA levels in VSMCs treated with hydrogen peroxide following LGALS3 knockdown; *n* = 3 per group. (N) RT-qPCR analysis of MMP9 and IL-1β mRNA levels in VSMCs transfected with mimic-NC or tRF-AspGTC mimic and si-NC or si-LGALS3, followed by hydrogen peroxide treatment; *n* = 3 per group. (O) WB analysis of CNN1, α-SMA, and MMP9 expression levels. (P) Quantitative analysis of (O); *n* = 3 per group. (Q) Detection of ROS generation. *P < 0.05; **P < 0.01; ***P < 0.001; ****P < 0.001. IOD, integrated optical density; si-LGALS3, small interfering RNA targeting lectin galactoside-binding soluble 3.

Based on the interaction between tRF-AspGTC and galectin-3 and its influence on galectin-3 expression, we hypothesize that tRF-AspGTC exerts its biological functions by targeting galectin-3. Initially, we observed elevated mRNA (Fig. 6A) and protein levels (Fig. 6C to E and G) of galectin-3 in clinical IA tissues. Similarly, increased mRNA (Fig. 6B) and protein levels (Fig. 6F and H) of galectin-3 were observed in the cerebral vascular tissues of mice with IA, indicating that galectin-3 may play an important role in the formation of IAs. To further investigate this, we constructed siRNA targeting galectin-3 and verified its knockdown efficiency (Fig. S5A to C). WB analysis demonstrated that galectin-3 knockdown could reverse hydrogen peroxide-induced phenotypic switching (Fig. 6I and J) of VSMCs and the expression of MMP9 (Fig. 6K and L). Additionally, galectin-3 knockdown significantly reduced the hydrogen peroxide-induced mRNA levels of MMP9 and IL-1β (Fig. 6M). To further confirm that tRF-AspGTC exerts its biological effects through its target gene galectin-3, we cotransfected VSMCs with tRF-AspGTC mimics and LGALS3 siRNA. The results showed that overexpression of tRF-AspGTC exacerbated hydrogen peroxide-induced VSMC phenotypic switching (Fig. 6O and P), MMP9 expression (Fig. 6N to P), IL-1β expression (Fig. 6N), and ROS production (Fig. 6Q). However, these effects induced by tRF-AspGTC were reversed upon galectin-3 knockdown, underscoring that galectin-3 is a downstream effector of tRF-AspGTC. In conclusion, tRF-AspGTC influences VSMC phenotypic switching, MMP9 expression, and oxidative stress and inflammatory responses by targeting galectin-3.

### tRF-AspGTC activates the TLR4/MyD88/NF-κB pathway through galectin-3

**Fig. 7.**
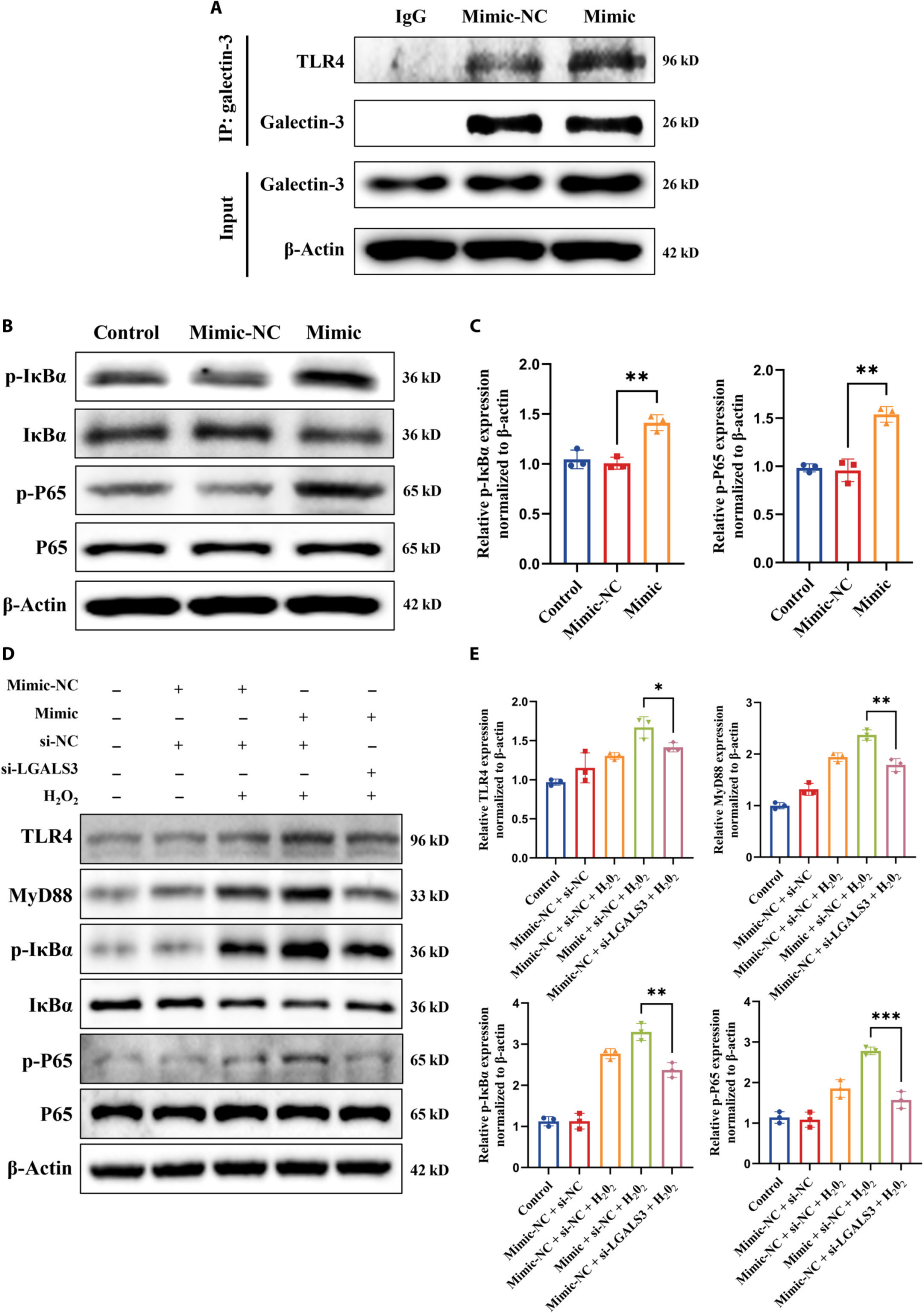
tRF-AspGTC activates the toll-like receptor 4 (TLR4)/MyD88/nuclear factor kappa B (NF-κB) pathway by stabilizing galectin-3 in VSMCs. (A) WB analysis of TLR4 immunoprecipitated with galectin-3 in VSMCs transfected with mimic-NC or tRF-AspGTC mimic. (B) WB analysis of NF-κB pathway proteins after tRF-AspGTC overexpression. (C) Quantitative analysis of (B); *n* = 3 per group. (D) WB analysis of TLR4/MyD88/NF-κB pathway proteins in VSMCs transfected with mimic-NC or tRF-AspGTC mimic and si-NC or si-LGALS3, followed by hydrogen peroxide treatment. (E) Quantitative analysis of (D); *n* = 3 per group. *P < 0.05; **P < 0.01; ***P < 0.001. IκBα, inhibitor of nuclear factor kappa-B alpha; p-IκBα, phosphorylated inhibitor of nuclear factor kappa-B alpha.

Galectin-3 is recognized as an endogenous paracrine ligand for TLR4 through its carbohydrate recognition domain [29,46]. Studies have indicated that the TLR4 pathway accelerates inflammation in arterial walls, thereby promoting the development and rupture of IA [47,48]. To further explore the mechanism by which galectin-3 activates inflammation in IA, we conducted protein immunoprecipitation experiments, confirming the specific binding of galectin-3 to TLR4 (Fig. 7A). Additionally, we observed enhanced binding between them upon overexpression of tRF-AspGTC (Fig. 7A). Upon activation, TLR4 can signal through MyD88-dependent and TRIF-dependent pathways, activating parallel signaling cascades, including those of NF-κB and mitogen-activated protein kinase, which activate transcription factors that regulate the production of pro-inflammatory cytokines [49–51]. We first examined the activation status of the downstream pathways of TLR4 following tRF-AspGTC overexpression in VSMCs under physiological conditions. The results revealed activation of the NF-κB pathway upon tRF-AspGTC overexpression, as evidenced by a marked increase in the phosphorylation of inhibitor of nuclear factor kappa-B alpha (IκBα) and P65 (Fig. 7B and C), while the mitogen-activated protein kinase pathway remained inactive (Fig. S6A and B). This suggests that tRF-AspGTC may trigger inflammation through NF-κB pathway activation. To further confirm these findings, we transfected tRF-AspGTC mimics and LGALS3 siRNA under pathological conditions. Overexpression of tRF-AspGTC significantly enhanced the expression of TLR4 induced by hydrogen peroxide, as well as the activation of downstream pathways involving MyD88 and NF-κB. Interestingly, knocking down LGALS3 suppressed the activation of these pathways (Fig. 7D and E). In conclusion, tRF-AspGTC activates the TLR4/MyD88/NF-κB pathway through galectin-3.

### Knockdown of tRF-AspGTC inhibits IA formation and progression in vivo

**Fig. 8.**
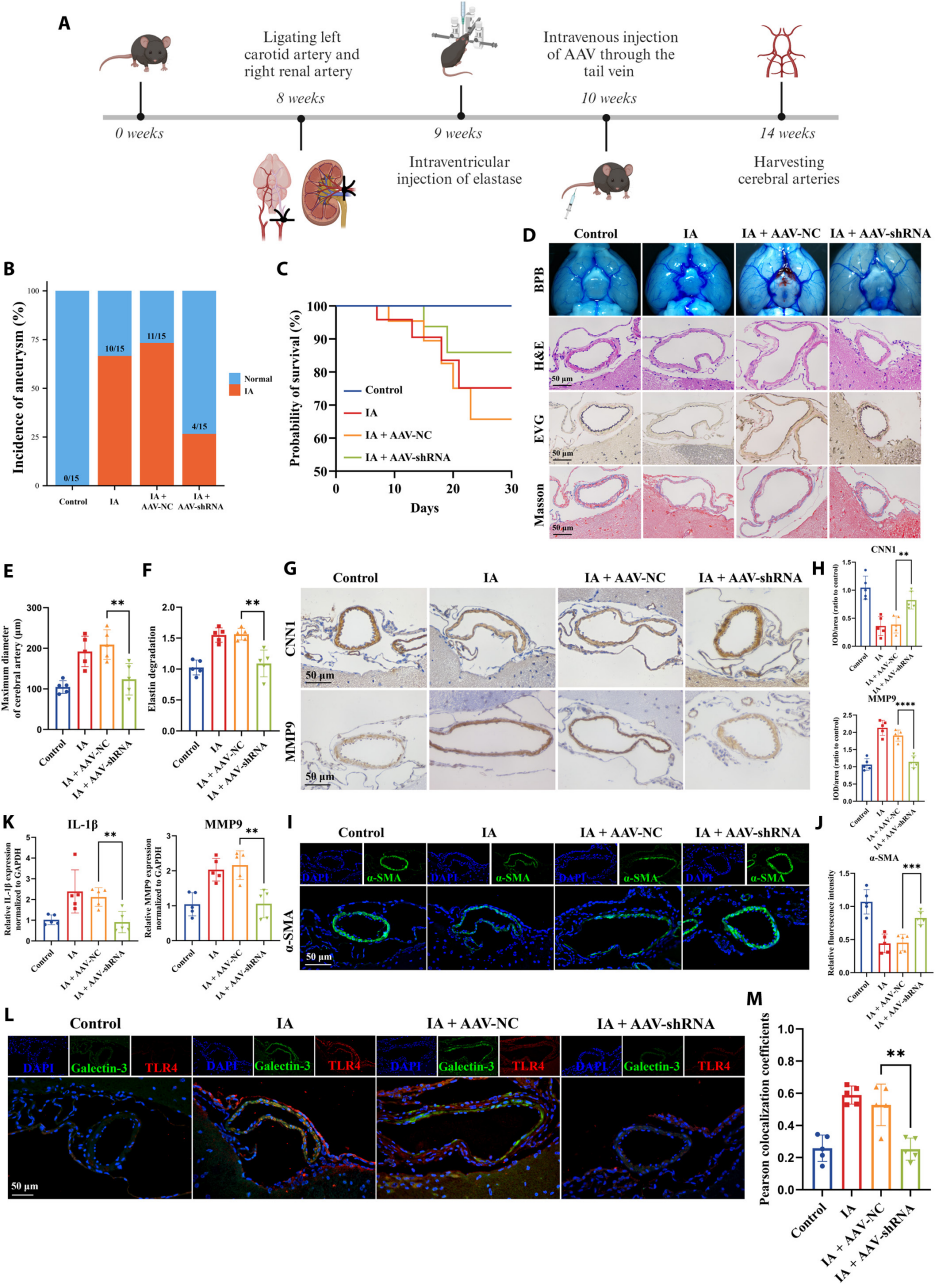
Knockdown of tRF-AspGTC inhibits IA formation and progression in vivo. (A) Schematic diagram of the animal model construction process. (B) Incidence of IAs in the indicated groups. (C) Survival curves of mice in the indicated groups. (D) Representative images of bromophenol blue perfusion staining, hematoxylin and eosin (H&E) staining, elastica–Van Gieson (EVG) staining, and Masson’s trichrome staining of the circle of Willis in mice. (E) Quantitative analysis of the maximum diameter of cerebral arteries in mice across the indicated groups; *n* = 5 per group. (F) Quantitative analysis of relative elastin degradation in the cerebral arteries of mice across the indicated groups; *n* = 5 per group. (G) IHC staining of CNN1 and MMP9 in the cerebral arteries of mice across the indicated groups. (H) Quantitative analysis of (G); *n* = 5 per group. (I) IF staining of α-SMA in cerebral arteries of mice across the indicated groups. (J) Quantitative analysis of (I); *n* = 5 per group. (K) RT-qPCR analysis of MMP9 and IL-1β mRNA expression levels in the cerebral arteries of mice across the indicated groups; *n* = 5 per group. (L) IF staining of galectin-3 and TLR4 in the cerebral arteries of mice across the indicated groups. (M) Pearson colocalization coefficient analysis of (L); *n* = 5 per group. **P < 0.01; ***P < 0.001; ****P < 0.0001. AAV, adeno-associated virus; shRNA, short hairpin RNA; BPB, bromophenol blue.

To evaluate the impact of tRF-AspGTC on IA formation and progression in vivo, we established an IA mouse model by intraventricular injection of elastase in hypertensive mice (left carotid artery ligation + right renal artery ligation). Additionally, we constructed recombinant adeno-associated virus 9 (AAV9) vectors encoding short hairpin RNA (shRNA) targeting tRF-AspGTC and administered them to mice via tail vein injection (Fig. 8A). A notable reduction in IA incidence (Fig. 8B) and an improvement in survival rate (Fig. 8C) were observed in mice following tRF-AspGTC interference. Subsequent analysis of mouse cerebral arteries revealed that IA mice exhibited enlarged intracranial artery diameters, elastin fiber degradation observed via elastica–Van Gieson staining, and collagen fiber disorder indicated by Masson staining. These events were reversed in mice receiving AAV-shRNA–tRF-AspGTC treatment compared to those treated with an empty vector (Fig. 8D to F). Immunohistochemical (IHC) (Fig. 8G and H) and IF (Fig. 8I and J) demonstrated increased expression of CNN1 and α-SMA in IA mice following AAV-shRNA–tRF-AspGTC treatment, suggesting alleviation of VSMC phenotypic switching in vivo. Additionally, IHC (Fig. 8G and H) revealed that AAV-shRNA–tRF-AspGTC treatment led to a decrease in MMP9 protein levels in IA mice, while RT-qPCR results (Fig. 8K) indicated that this treatment reduced MMP9 and IL-1β expression levels. Finally, IF (Fig. 8L and M) revealed increased expression of galectin-3 and TLR4 in the cerebral arteries of IA mice, whereas their expression was attenuated following treatment with AAV-shRNA–tRF-AspGTC. The above findings suggest that tRF-AspGTC promotes the occurrence of IA by inducing VSMC phenotypic switching, MMP9 expression, and inflammation through the galectin-3/TLR4 pathway.

## Discussion

This study provides the first detailed exploration of the role of tsRNA in the pathogenesis of IA. Initially, we identified differentially expressed tsRNAs in patients with IA through high-throughput sequencing of circulating exosomes. Subsequently, we validated the most significantly up-regulated tsRNA, tRF-AspGTC, in both human and murine IA tissues. We then performed gain- and loss-of-function experiments in VSMCs, revealing that tRF-AspGTC promotes VSMC phenotypic switching, up-regulates MMP9 and IL-1β expression, and increases ROS production. Furthermore, using RNA pulldown and mass spectrometry analyses, we discovered that tRF-AspGTC specifically binds to galectin-3 and stabilizes it by inhibiting TRIM29-mediated ubiquitination and degradation. This stabilization activates the downstream TLR4/MyD88/NF-κB pathway, thereby promoting the development of IAs in vivo. Additionally, ROC analysis revealed an area under the curve of 0.816, indicating that circulating exosomal tRF-AspGTC has good diagnostic efficacy for IA. Multivariate logistic regression analysis identified the expression level of tRF-AspGTC as an independent risk factor for the occurrence of IA. These findings indicate that tRF-AspGTC is a critical factor in the formation of IA and holds promise as both a diagnostic biomarker and a therapeutic target.

tsRNAs are small, noncoding RNAs derived from precursor or mature tRNAs by specific endonucleases in response to oxidative stress, hypoxia, or other adverse conditions [18,52]. As one of the oldest small RNA species, tsRNAs exhibit widespread and stable expression, with a high level of evolutionary conservation across different species [53]. tsRNAs are crucial in mRNA silencing, translational regulation, cell apoptosis, necrosis, and intercellular communication, and they are strongly associated with the development of various diseases [52,54–56]. tsRNAs can achieve endogenous gene silencing through a mechanism similar to that of microRNAs by binding complementarily to the 3′ untranslated region of target mRNA molecules [40,57]. Compared to other typical small noncoding RNAs, tsRNAs exhibit a broader range of mechanisms [20,58–60]. Research has reported that tsRNAs can directly interact with RBPs to promote tumor development [24]. In our research, we found that tRF-AspGTC can directly bind to galectin-3 and influence its ubiquitination modification. This represents the first discovery in VSMCs that tsRNAs can directly interact with RBPs to regulate disease progression.

The initiation and progression of IA involve critical pathological processes, including vascular wall remodeling, inflammatory response activation, and ECM degradation [6,39]. VSMCs are the primary cell type in the tunica media of intracranial arteries and play an essential role in maintaining the integrity of the cerebrovascular system [61]. Following vascular injury, VSMCs undergo phenotypic switching from a contractile (differentiated) to a synthetic (dedifferentiated) state [36,62,63]. Subsequently, the high expression of MMPs in synthetic VSMCs leads to ECM degradation [64]. Concurrently, oxidative stress and the activation of inflammatory responses accelerate vascular wall degradation, resulting in IA formation and rupture [65]. Research has demonstrated that epigenetic factors are pivotal in the phenotypic switching of VSMCs in IA [66–68]. In our research, we found that targeted silencing of tRF-AspGTC inhibits VSMC phenotypic switching both in vivo and in vitro, suggesting its key role in the underlying mechanisms of this process. MMP2 and MMP9, as gelatinases, are essential in degrading major components of the vascular ECM and internal elastic lamina [69]. Our findings indicated that tRF-AspGTC can regulate MMP9 expression without affecting MMP2 expression. Studies have linked the activation of the NF-κB pathway and the expression of the pro-inflammatory cytokine IL-1β to the up-regulation and activation of MMP9 [70]. In our study, tRF-AspGTC enhanced the stability of galectin-3 and activated the TLR4/MyD88/NF-κB pathway, thereby promoting IL-1β expression and significantly influencing MMP9 expression. Although MMP2 also plays a significant role in elastin degradation within aneurysms, tRF-AspGTC does not appear to be involved in the pathways regulating MMP2 expression and activation. The specific mechanisms of MMP2 expression and activation in IA require further investigation.

Unruptured IAs typically lack clinical symptoms; however, their rupture leading to SAH often results in poor clinical outcomes [2]. Therefore, early identification and intervention of IAs are crucial to preventing aneurysm development and rupture. Currently, the diagnosis of IAs primarily relies on invasive vascular imaging techniques such as computed tomography angiography and digital subtraction angiography, which are not practical for large-scale population screening [10]. Thus, identifying blood biomarkers with high sensitivity and specificity is essential for developing cost-effective, population-wide screening methods for detecting IAs. Exosomes actively secreted by various cell types into peripheral blood, including VSMCs, endothelial cells, and mesenchymal stem cells, provide a better reflection of biological changes within vascular lesions [71]. The protective role of exosome membranes reduces enzymatic degradation of molecules in bodily fluids, significantly increasing the stability of exosome contents [72–74]. Therefore, we expanded our study cohort to examine the tRF-AspGTC expression levels in circulating exosomes from patients with IA. ROC analysis revealed that tRF-AspGTC in circulating exosomes exhibits good sensitivity and specificity, making it a valuable clinical diagnostic marker for IAs.

This study has some limitations. Due to factors such as a limited sample size and ethnic variations, further validation of the diagnostic effectiveness of circulating exosome tRF-AspGTC is needed in an independent, prospective IA cohort.

**Fig. 9.**
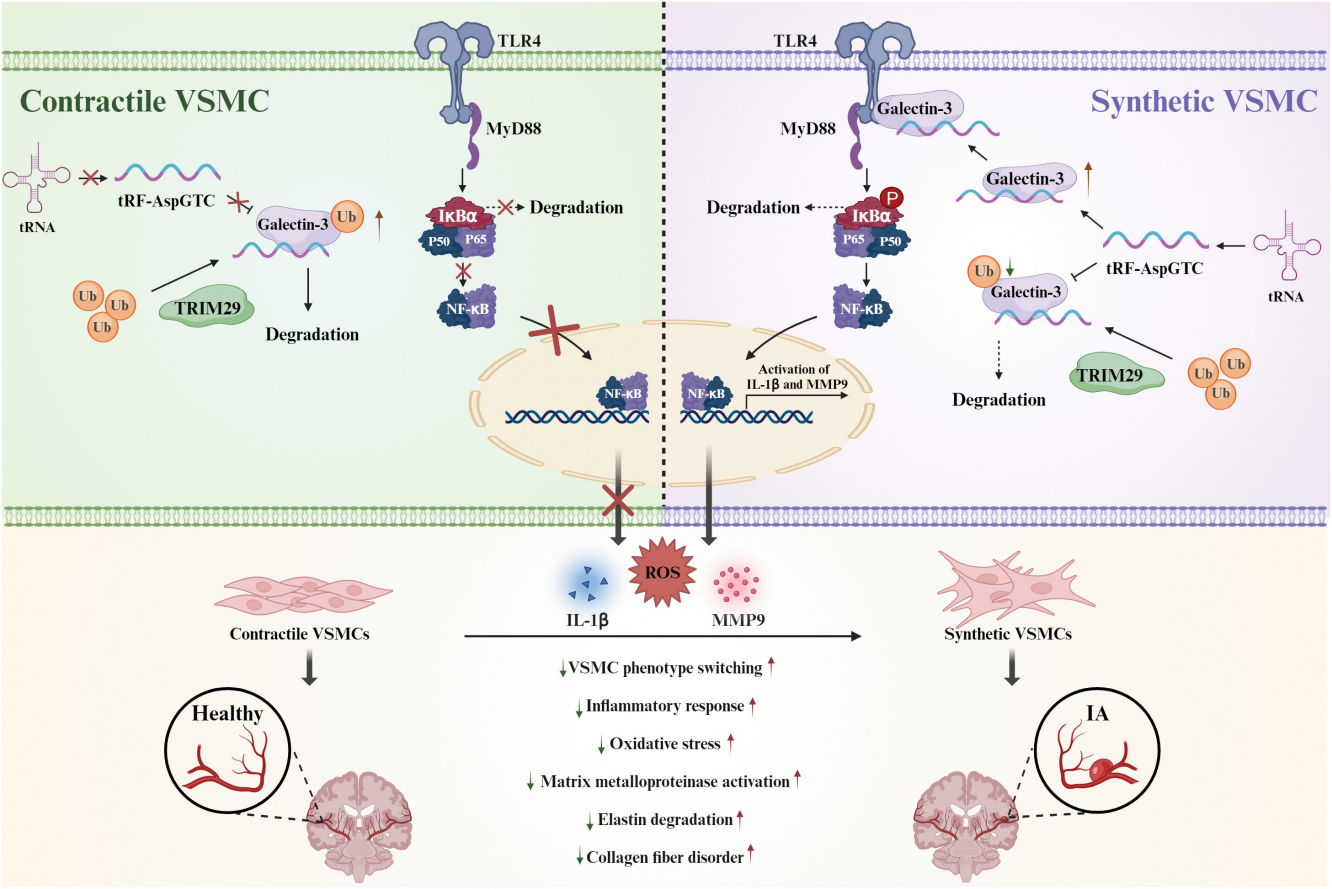
Proposed model of tRF-AspGTC in the pathogenesis of IA. tRF-AspGTC binds to galectin-3, inhibiting TRIM29-mediated ubiquitination and stabilizing galectin-3. This stabilization activates the TLR4/MyD88/NF-κB pathway, leading to inflammatory responses, oxidative stress, activation of MMPs, and phenotypic switching of VSMCs, ultimately culminating in the formation of IA. tRNA, transfer RNA; Ub, ubiquitin.

In summary, our study demonstrates that tRF-AspGTC can suppress the ubiquitination of galectin-3 mediated by TRIM29, thereby enhancing its stability and activating the TLR4/MyD88/NF-κB signaling pathway. This activation promotes the phenotypic switching of VSMCs, MMP9 expression, and oxidative stress and inflammatory responses, ultimately contributing to the formation of IAs (Fig. 9). Our findings highlight the significance of tsRNAs in IAs and suggest that tRF-AspGTC has potential as a valuable diagnostic biomarker and a novel therapeutic target for IAs.

## Methods

### Human samples

The human research protocol was approved by the Research Ethics Committee of the Affiliated Hospital of Qingdao University, following the guidelines outlined in the Helsinki Declaration (Approval Number: QYFYWZLL28320). Fresh human IA tissue samples were obtained from patients who underwent craniotomy for clipping surgery, while healthy STA tissues were taken from patients with cranial trauma for comparison. Blood samples were collected from patients diagnosed with unruptured IAs, with blood from healthy volunteers serving as controls. Informed consent was secured from all participants involved in the research.

### Exosome isolation

Plasma samples were initially centrifuged at 2,000 × *g* at 4 °C, followed by a second spin at 10,000 × *g*. The resulting supernatant was filtered through a 0.45-μm sterile membrane, and the pellet was resuspended in cold phosphate-buffered saline (PBS). Exosome purification was then achieved with a final centrifugation at 100,000 × *g* at 4 °C for 70 min, after which the pellet was resuspended in PBS.

### Animal experiments

The animal protocol used in this study was approved by the Ethics Committee of the Affiliated Hospital of Qingdao University, and the study followed the National Institutes of Health Guidelines for the Care and Use of Laboratory Animals (Approval Number: QYFYWZLL28320). Chronic hypertension was established in 8-week-old male C57BL/6 mice through ligation of the left carotid artery and the right renal artery. The following week, a small hole was drilled 1.2 mm anterior and 0.7 mm right of the bregma, through which elastase solution was slowly injected into the right basal cistern. The AAV9 serotype interfering with tRF-AspGTC expression was constructed and packaged by OBiO Technology (Shanghai) Corp., Ltd. The mice were randomly assigned to groups and given a single intravenous injection via the tail vein with 2 × 10^11^ vg of either AAV-shRNA–tRF-AspGTC or AAV-shRNA-NC (empty vector control). They were then continuously fed a high-salt (8% sodium chloride) and β-aminopropionitrile (0.12%) diet for 4 weeks.

### Cell culture and treatment

Human brain vascular smooth muscle cells were cultured in Dulbecco’s modified Eagle medium supplemented with 10% fetal bovine serum (Inner Mongolia Opcel Biotechnology Co., Ltd., China) and double antibiotics and maintained at 37 °C in a 5% CO_2_ atmosphere. We designed and synthesized mimics and inhibitors of tRF-AspGTC, as well as small interfering sequences targeting LGALS3 and TRIM29, through Shanghai GenePharma Co., Ltd. The sequences are shown in Table S2. When the cell density reached approximately 70%, transfection experiments were performed using Lipofectamine 3000 (Invitrogen, American).

### Reverse transcription quantitative polymerase chain reaction

RNA was extracted with TRIzol reagent (Bioflux, China) and then reverse transcribed into complementary DNA utilizing the SPARKscript II RT Plus Kit (with gDNA Eraser) (Shandong Sparkjade Biotechnology Co., Ltd., China) and miRNA 1st Strand cDNA Synthesis Kit (Yeasen Biotech Co., Ltd., China). Quantification of RNA expression levels was performed via RT-qPCR using SYBR Green qPCR Mix (Yeasen Biotech Co., Ltd., China) and a LightCycler 96 instrument (Roche, Switzerland). RNA expression was normalized using glyceraldehyde-3-phosphate dehydrogenase or U6 as internal reference genes. The 2^−ΔΔCT^ method was used to calculate the average data from 3 independent experiments, with primer sequences detailed in Table S3.

### Fluorescence in situ hybridization

The 3′-end Cy3 fluorescently labeled tRF-AspGTC was synthesized by GenePharma (Shanghai, China). The sequence is shown in Table S3. The distribution of tRF-AspGTC within VSMCs and IA tissue was assessed using the FISH kit (Shanghai GenePharma Co., Ltd., China). For this, cell slides or frozen tissue sections underwent permeabilization using 0.1% Triton X-100 at 25 °C for 15 min. Subsequently, a blocking solution was applied to inhibit nonspecific binding. The probe working solution was then prepared and allowed to incubate at 37 °C for 12 h. Finally, cell nuclei were stained with 4′,6-diamidino-2-phenylindole and observed using a confocal microscope.

### RNA pulldown and mass spectrometry analysis

The 3′ end biotinylated tRF-AspGTC probe and NC probe were synthesized by GenePharma (Shanghai, China). The sequence is shown in Table S3. VSMCs were lysed using a buffer containing an RNase inhibitor. Subsequently, streptavidin-coated agarose beads, coupled with the biotinylated probes, were introduced into the cell lysates and allowed to incubate overnight at 4 °C with gentle rotation. The RNA–protein complexes, now bound to the beads, were then released using lysis buffer, followed by separation of proteins using sodium dodecyl sulfate polyacrylamide gel electrophoresis. Protein bands extracted from the gel were analyzed via mass spectrometry by Shanghai Bioprofile Technology Co., Ltd [75].

### Western blot

Treated cells were lysed in radioimmunoprecipitation assay buffer (Solarbio, China) with phenylmethylsulfonyl fluoride on ice for 30 min. Samples were combined with loading buffer and heated at 98 °C for 10 min. They were then separated by 10% sodium dodecyl sulfate polyacrylamide gel electrophoresis and transferred to polyvinylidene fluoride membranes. After blocking, the membrane was incubated with the primary antibody overnight at 4 °C: anti-MHC (K002095P, Solarbio, China), anti-CNN1 (abs171608, Absin, China), anti-α-SMA (BM0002, Boster, China), anti-galectin-3 (60207-1-Ig, Proteintech, China), anti-TRIM29 (17542-1-AP, Proteintech, China), anti-MMP9 (BM4089, Boster, China), anti-MMP2 (A00286-2, Boster, China), anti-ubiquitin (#3936, Cell Signaling Technology, America), anti-TLR4 (66350-1-Ig, Proteintech, China), anti-MyD88 (23230-1-AP, Proteintech, China), anti-IκBα (abs131168, Absin, China), anti-phosphorylated inhibitor of nuclear factor kappa-B alpha (abs172314, Absin, China), anti-P65 (BF8005, Affinity, China), anti-p-P65 (bs-0271R, BIOSS, China), and anti-β-actin (66009-1-Ig, Proteintech, China). After washing, membranes were incubated with horseradish peroxidase-conjugated secondary antibody for 1 h. Signals were detected using a chemiluminescence imaging system (Millipore, USA) with the lumiQ Universal ECL Substrate (SB-WB012, Share-bio, Shanghai) and quantified using the ImageJ software, normalized to β-actin levels.

### Histological assay

The Willis circle of mouse cerebral arteries was excised and fixed in 4% paraformaldehyde. Afterward, the specimens underwent dehydration using an ethanol gradient, embedding in paraffin, and sectioning into 6-μm tissue slices. Following this, tissue samples were stained according to the instructions using hematoxylin and eosin, elastica–Van Gieson, or Masson’s trichrome staining for histological analysis.

### IF and IHC staining

For IF staining, mouse brain artery sections were initially fixed and subsequently permeabilized. They were then incubated in 1% bovine serum albumin for 30 min before being exposed to primary antibodies (anti-α-SMA, anti-galectin-3, and anti-TLR4) overnight at 4 °C. After 3 washes with PBS with Tween 20, the sections were incubated with fluorescein isothiocyanate–conjugated AffiniPure Goat Anti-Mouse IgG (SA00003-1, Proteintech, China) and Cy3-conjugated AffiniPure Goat Anti-Rabbit IgG (SA00009-2, Proteintech, China) for 1 h at room temperature. Fluorescence signals were visualized using a fluorescence microscope.

For IHC staining, paraffin sections of human/mouse arteries underwent dewaxing/rehydration steps and were treated with 3% hydrogen peroxide before antigen retrieval in sodium citrate buffer (95 °C). Following blocking with 1% bovine serum albumin at 37 °C, the sections were incubated overnight at 4 °C with primary antibodies (anti-galectin-3, anti-CNN1, and anti-MMP9) and then exposed to secondary antibodies. Sections were then stained with diaminobenzidine and counterstained with hematoxylin.

### Coimmunoprecipitation assays

The cellular lysate supplemented with protease inhibitors was incubated with anti-galectin-3 (60207-1-Ig, Proteintech, China) or immunoglobulin G antibody, along with magnetic beads, at 4 °C for 12 h. Subsequently, the magnetic beads were washed with lysis buffer. Finally, proteins bound to the magnetic beads were eluted for WB analysis.

### ROS detection

Following the instructions of the ROS Detection Kit (Solarbio, China), the 2′,7′-dichlorodihydrofluorescein diacetate probe was diluted and incubated with the cells at 37 °C in a cell culture incubator for 30 min. Finally, the cells were observed and imaged using a fluorescence microscope.

### Protein–RNA docking

Protein–RNA docking was performed to predict the interaction between tRF-AspGTC and galectin-3. The galectin-3 sequence (UniProt ID: P17931) was retrieved from the UniProt database (https://www.uniprot.org). The RNA sequence of tRF-AspGTC was obtained from our experimental data. Docking was carried out using the HDOCK server (http://hdock.phys.hust.edu.cn/), which integrates template-based and free docking methods to predict protein–RNA interactions [76].

### Statistical analysis

All data are presented as mean ± standard deviation (SD). Statistical analyses were performed using SPSS version 27.0. For statistical comparisons, we first assessed the normality of the data and the equality of variances. A 2-tailed, unpaired or paired Student *t* test was employed to evaluate statistical differences between 2 groups. If equal variances were not assumed, the Welch *t* test was applied. For data not normally distributed, the nonparametric Mann–Whitney *U* test was used to compare 2 samples. Differences among multiple groups were analyzed using analysis of variance followed by Tukey’s multiple comparison test. Pearson correlation analysis was conducted to assess the correlation between the expression levels of 2 genes. Survival rates were estimated using the Kaplan–Meier method. Diagnostic performance was evaluated using the ROC curve. Clinical independent risk factors were analyzed using multivariate logistic regression analysis. A *P* value of <0.05 was considered statistically significant.

## Data Availability

The data of this study are available from the corresponding authors on reasonable request.
