# Supplementary material for: tRF-AspGTC Promotes Intracranial Aneurysm Formation by Controlling TRIM29-Mediated Galectin-3 Ubiquitination
Source: Research (Wash D C). 2025 Jan 7;8:0574. doi: 10.34133/research.0574 (PMC11704088; doi:10.34133/research.0574)
Supplement: Supplementary 1 — Figs. S1 to S6 Tables S1 to S3 [file research.0574.f1.docx]

**Table S1:** Multivariate regression analysis

|  | **Control** | **IA** | **Univariate  analysis** | | **Multivariate analysis** | |
| --- | --- | --- | --- | --- | --- | --- |
|  |  |  | **P value** | **OR value (95% CI)** | **P value** | **OR value (95% CI)** |
| **Mean age (years)** | 59.6 ± 7.1 | 61.5 ± 6.3 | 0.151 | 1.045 (0.984 - 1.109) | - | - |
| **Gender** |  |  | 0.840 | 1.085 (0.491 - 2.395) |  |  |
| Male | 22 | 21 |  |  |  |  |
| Female | 28 | 29 |  |  |  |  |
| **Hypertension** |  |  | 0.058 | 2.633 (0.967 - 7.170) | 0.065 | 2.937 (0.933 - 9.242) |
| Yes | 7 | 15 |  |  |  |  |
| No | 43 | 35 |  |  | - | - |
| **Diabetes mellitus** |  |  | 0.280 | 1.833 (0.611 - 5.502) |  |  |
| Yes | 6 | 10 |  |  |  |  |
| No | 44 | 40 |  |  |  |  |
| **Drinking** |  |  | 0.618 | 1.285 (0.480 - 3.437) |  |  |
| Yes | 9 | 11 |  |  |  |  |
| No | 41 | 39 |  |  |  |  |
| **Smoking** |  |  | 0.649 | 0.812 (0.332 - 1.989) |  |  |
| Yes | 14 | 12 |  |  |  |  |
| No | 36 | 38 |  |  |  |  |
| **tRF-AspGTC** | 1.12±0.55 | 1.99±0.98 | < 0.001 | 6.325 (2.671 - 14.978) | < 0.001 | 6.736 (2.767 - 16.397) |

**Table S2:** tRF-AspGTC inhibitor, mimic and siRNA were used in this study.

|  | **Sequences (5’-3’)** |
| --- | --- |
| inhibitor-NC | CAGUACUUUUGUGUAGUACAA |
| tRF-AspGTC-inhibitor | GCUCCCCGUCGGGGAAUUGAACCCCGGUCUCCCGC |
| mimic-NC | CAACGUGACACGUUCGGAGAA |
| tRF-AspGTC-mimic | GCGGGAGACCGGGGUUCAAUUCCCCGACGGGGAGC |
| siRNA-NC | Sense: UUCUCCGAACGUGUCACGUTT  Antisense: ACGUGACACGUUCGGAGAATT |
| siRNA-LGALS3 | Sense: CACGCUUCAAUGAGAACAATT  Antisense: UUGUUCUCAUUGAAGCGUGTT |
| siRNA-TRIM29 | Sense: GUGCAUUGAUGAGCAAUUATT  Antisense: UAAUUGCUCAUCAAUGCACTT |

**Table S3:** Primers and probes were used in this study.

|  | **Sequences (5’-3’)** |
| --- | --- |
| Primer-tRF-AspGTC | ATTCCCCGACGGGGAGCAA |
| Primer-tRF-SerGCT | CGCGAGAAAGCTCACAAGAACTA |
| Primer-tRF-LeuCAG | CCTGGAGGCGTGGGTTCGA |
| Primer-tRF-GluCTC | GTGGTTAGGATTCGGCGCTAA |
| Primer-tRF-GlyGCC | TGGTTCAGTGGTAGAATTCTCGC |
| Primer-tRF-LeuCAA | AATGGAGGCGTGGGTTCGAAA |
| The reverse primers of all the small RNA were used the mRQ 3’ universal primer and U6 primers were provided by manufacturer. | |
| Primer-GAPDH (Human) | F：AAGAAGGTGGTGAAGCAGGC  R：TCCACCACCCAGTTGCTGTA |
| Primer-LGALS3 (Human) | F：CCATCTTCTGGACAGCCAAGTG  R：TATCAGCATGCGAGGCACCACT |
| Primer-TRIM29 (Human) | F：GCACCGGACACCATGAAGA  R：GACATAGAATGGCCGGTAGTG |
| Primer-MMP9 (Human) | F：CCTGGAGACCTGAGAACCAA  R：AGTGTAACCATAGCGGTACAGG |
| Primer-MMP2 (Human) | F：GTGAAGTATGGGAACGCCG  R：GCCGTACTTGCCATCCTTCT |
| Primer-IL-1β (Human) | F：ATGATGGCTTATTACAGTGGCAA  R：GTCGGAGATTCGTAGCTGGA |
| Primer-TNF-α (Human) | F：CCTCTCTCTAATCAGCCCTCTG  R：GAGGACCTGGGAGTAGATGAG |
| Primer-IL-6 (Human) | F：ACTCACCTCTTCAGAACGAATTG  R：CCATCTTTGGAAGGTTCAGGTTG |
| Primer-GAPDH (Mouse) | F：AGGTCGGTGTGAACGGATTTG  R：TGTAGACCATGTAGTTGAGGTCA |
| Primer-LGALS3 (Mouse) | F：AACACGAAGCAGGACAATAACTGG  R：GCAGTAGGTGAGCATCGTTGAC |
| Primer-MMP9 (Mouse) | F：GCAGAGGCATACTTGTACCG  R：TGATGTTATGATGGTCCCACTTG |
| Primer-IL-1β (Mouse) | F：GAAATGCCACCTTTTGACAGTG  R：TGGATGCTCTCATCAGGACAG |
| RNA pulldown probe-NC | CGGAGUGUGAGUAGGCUACUCGGGCGCACGCAGGU |
| RNA pulldown probe-tRF-AspGTC | GCGGGAGACCGGGGUUCAAUUCCCCGACGGGGAGC |
| FISH probe-NC | ACTGGCCCCCGGTCCGCAAGCGGCCATGTCTCTGC |
| FISH probe-tRF-AspGTC | GCTCCCCGTCGGGGAATTGAACCCCGGTCTCCCGC |

**
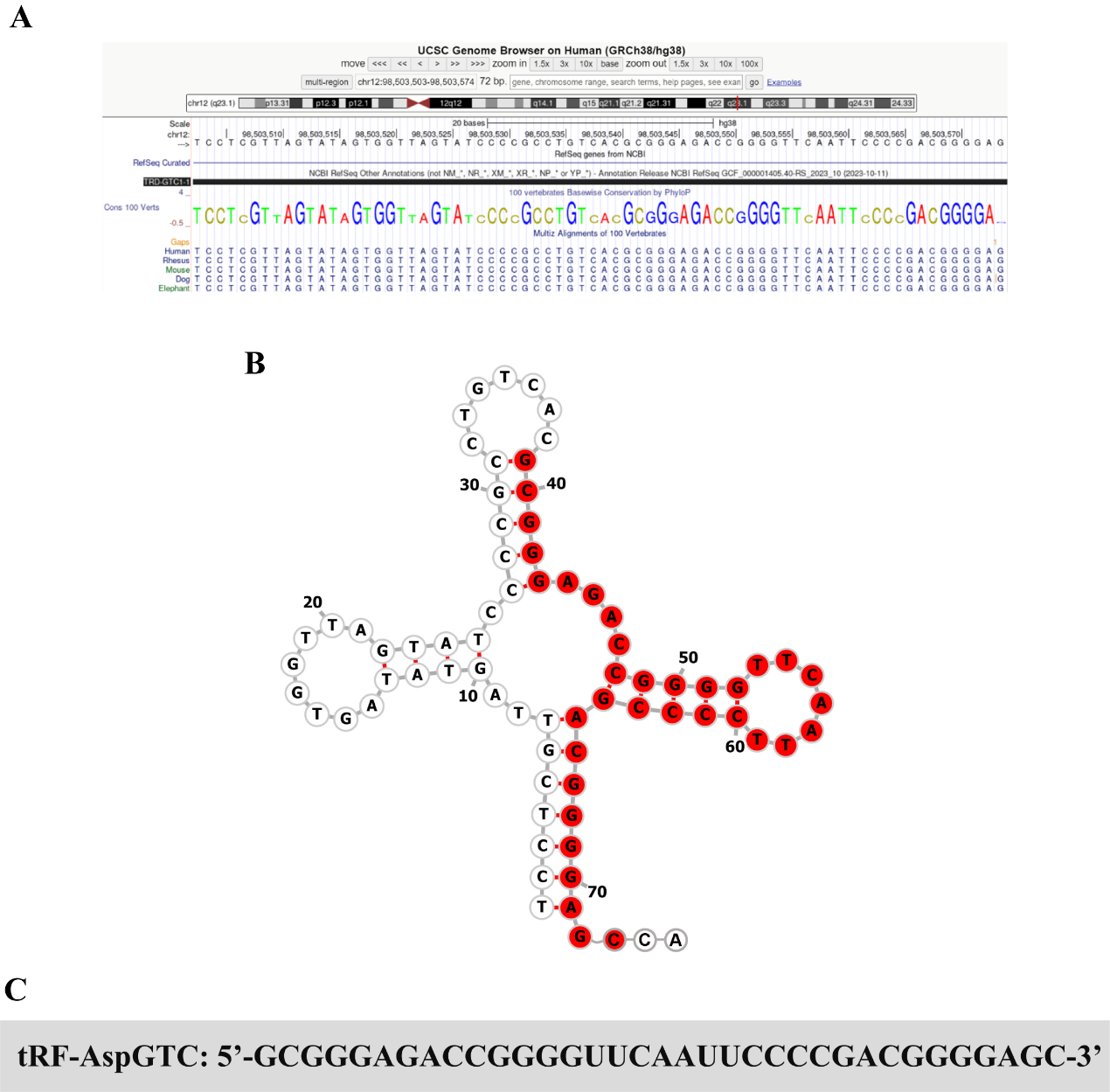
**

**Figure S1: Characteristics of tRF-AspGTC**

(A) The gene sequence and species conservation of tRNA-AspGTC as displayed in the UCSC Genome Browser. (B) tRF-AspGTC is derived from the tRNA-AspGTC. (C) The gene sequence of tRF-AspGTC.

**
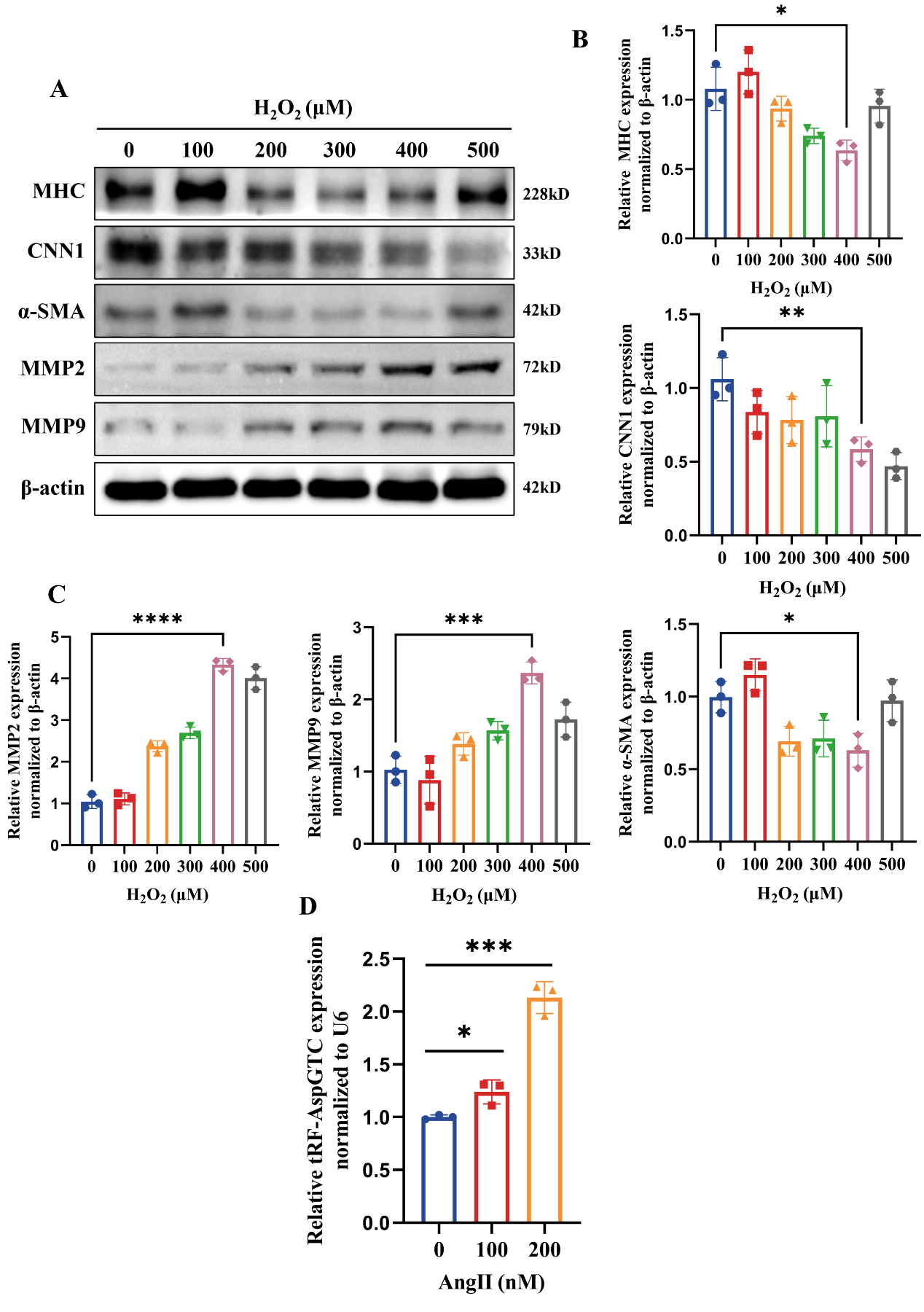
 Figure S2: Hydrogen peroxide can induce VSMCs phenotypic switching and MMPs expression**

(A) WB analysis of MHC, CNN1, α-SMA, MMP2, and MMP9 expression levels in VSMCs treated with different concentrations of hydrogen peroxide for 24 hours. (B) and (C) Quantitative analysis of Figure B; n=3 per group. (D) RT-qPCR analysis of tRF-AspGTC expression levels in VSMCs treated with different concentrations of Ang II for 24 hours; n=3 per group. *P<0.05, **P<0.01, ***P<0.001, ****P<0.0001


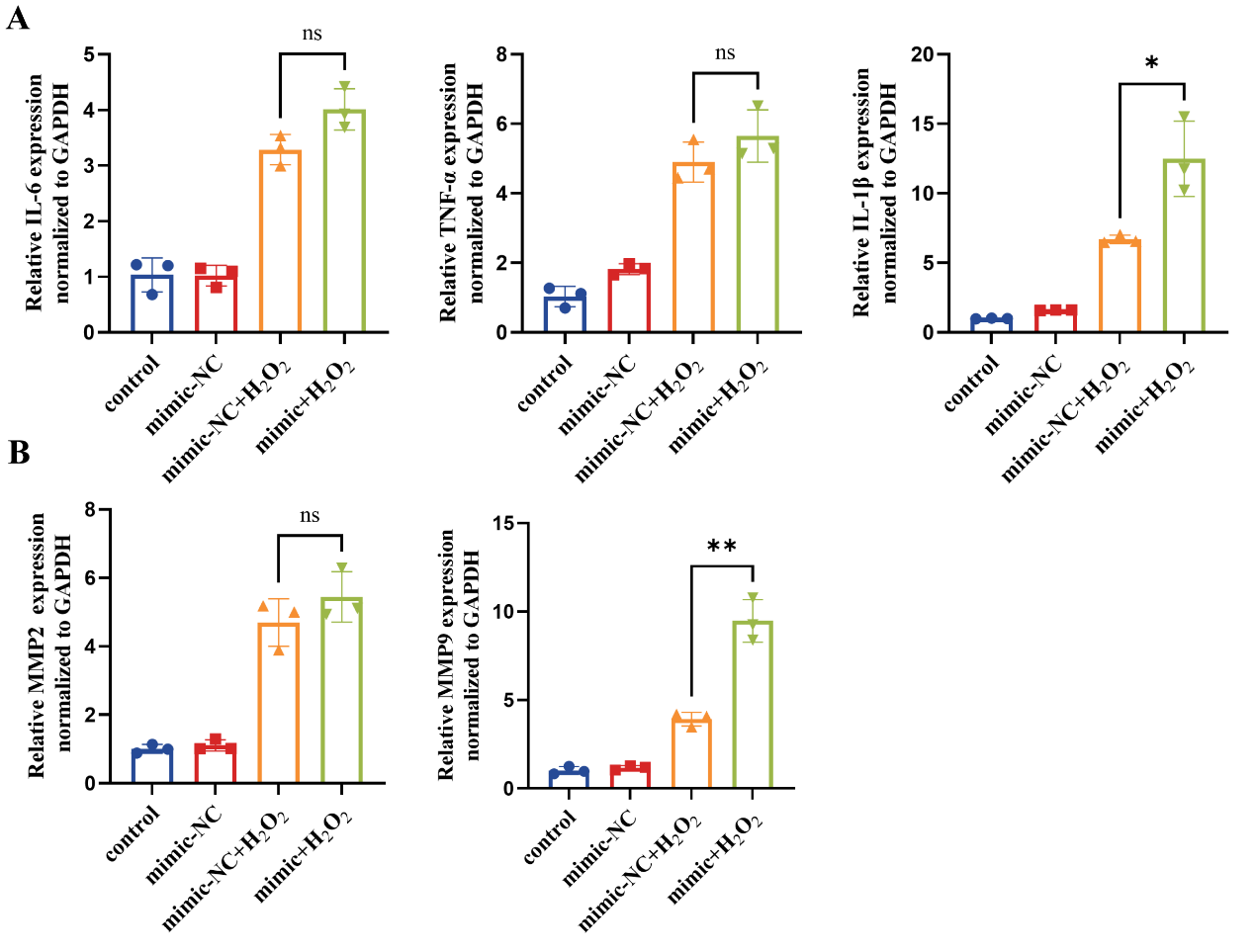


**Figure S3: tRF-AspGTC can exacerbate hydrogen peroxide-induced expression of MMP9 and IL-1β**

(A) RT-qPCR analysis of inflammatory cytokines (IL-6, TNF-α, and IL-1β) mRNA levels in VSMCs after overexpressing tRF-AspGTC and treating with hydrogen peroxide; n=3 per group. (B) RT-qPCR analysis of MMP2 and MMP9 mRNA levels in VSMCs after overexpressing tRF-AspGTC and treating with hydrogen peroxide; n=3 per group. *P<0.05, **P<0.01

**
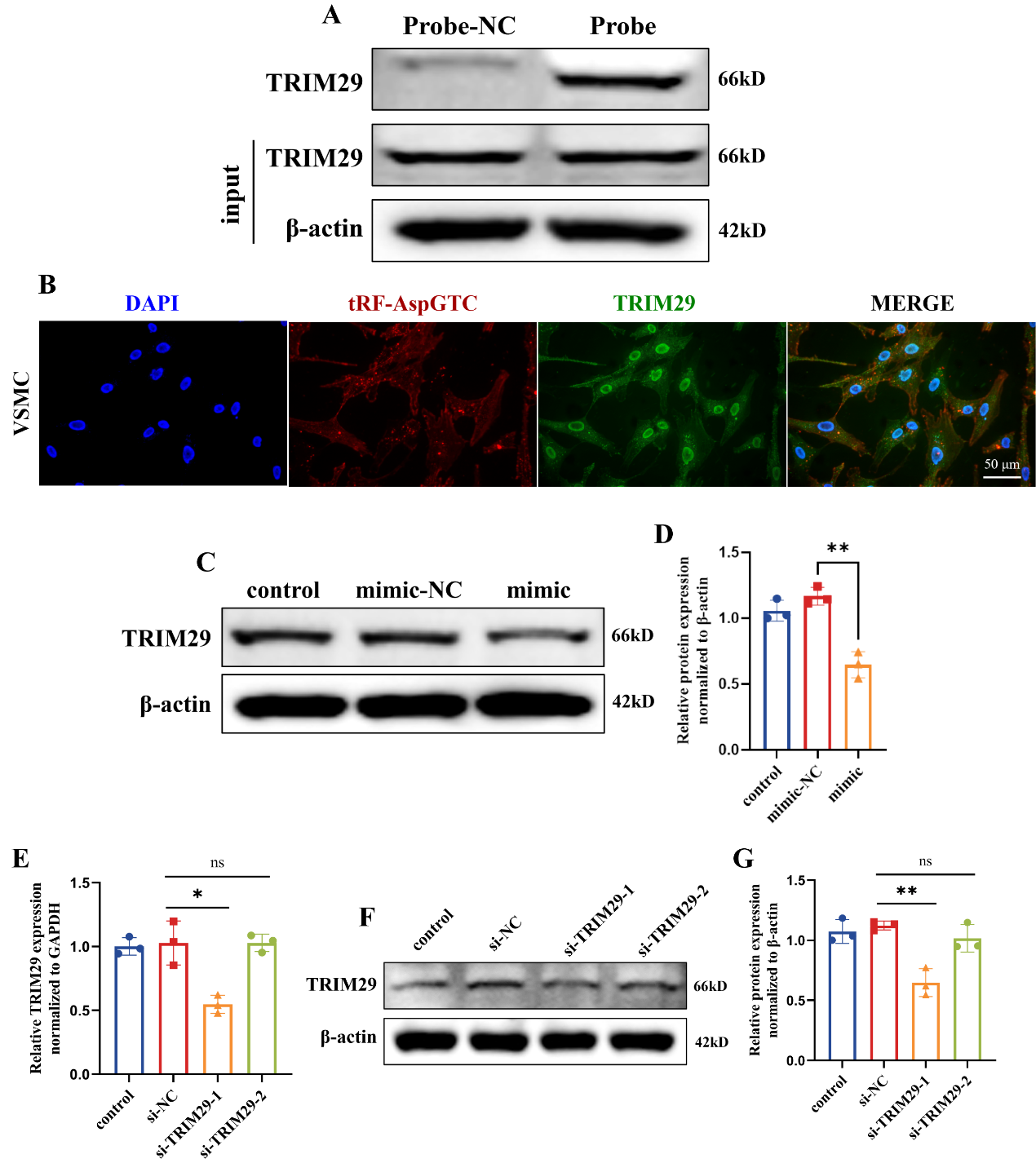
 Figure S4: tRF-AspGTC binds to TRIM29 and suppresses its expression.**

(A) Screening of tRF-AspGTC-specific binding proteins through RNA pulldown followed by WB detection of TRIM29. (B) Co-localization detection of tRF-AspGTC (red) and TRIM29 (green) via FISH and IF. (C) WB analysis of TRIM29 expression after tRF-AspGTC overexpression. (D) Quantitative analysis of Figure C; n=3 per group. (E) RT-qPCR analysis of TRIM29 knockdown efficiency in VSMCs transfected with si-TRIM29; n=3 per group. (F) WB analysis of TRIM29 knockdown efficiency in VSMCs transfected with si-TRIM29. (G) Quantitative analysis of Figure F; n=3 per group. *P<0.05, **P<0.01

**
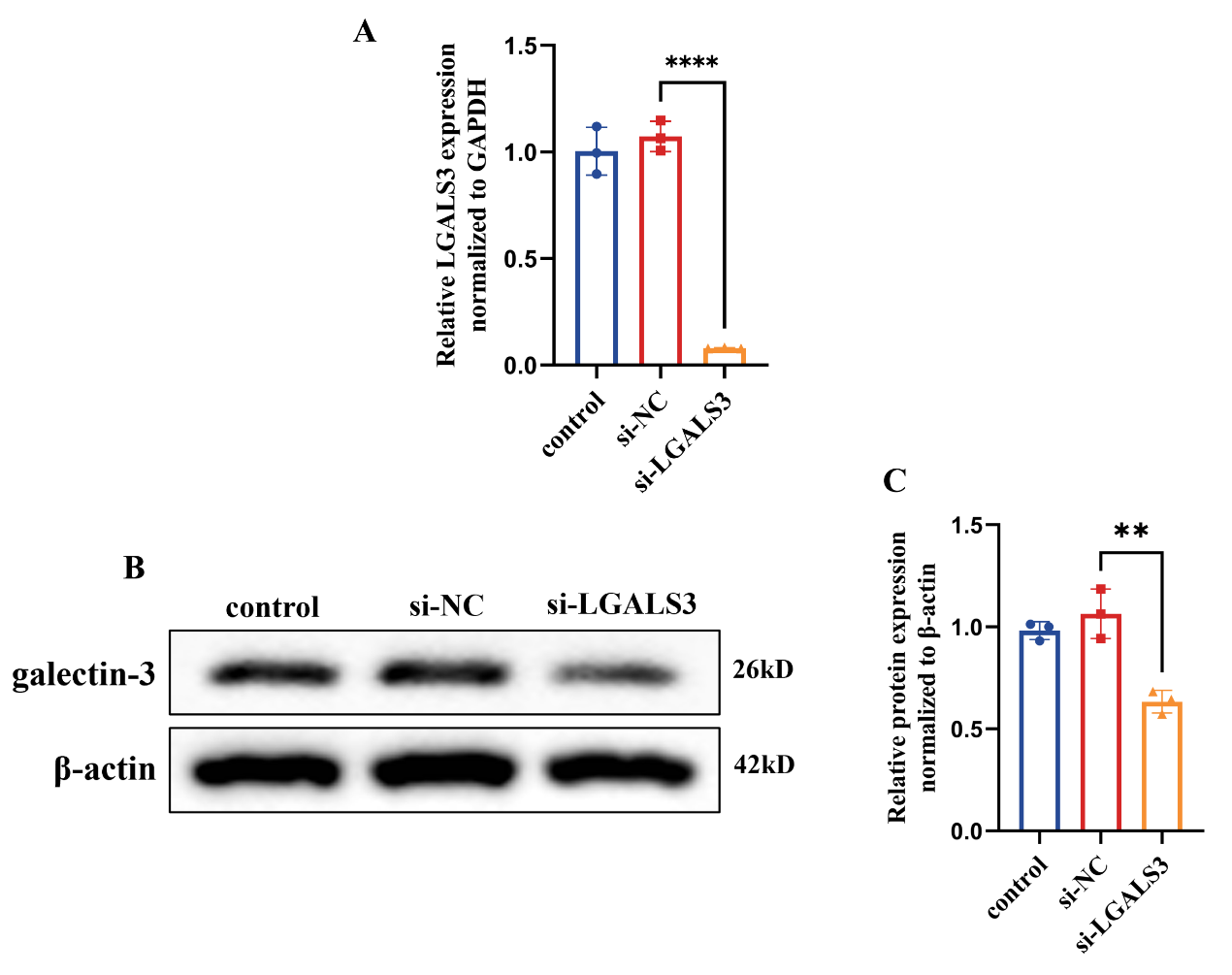
 Figure S5: Knockdown efficiency of LGALS3**

(A) RT-qPCR analysis of LGALS3 knockdown efficiency in VSMCs transfected with si-LGALS3; n=3 per group. (B) WB analysis of galectin-3 knockdown efficiency in VSMCs transfected with si-LGALS3. (C) Quantitative analysis of Figure B; n=3 per group. **P<0.01, ****P<0.0001

**
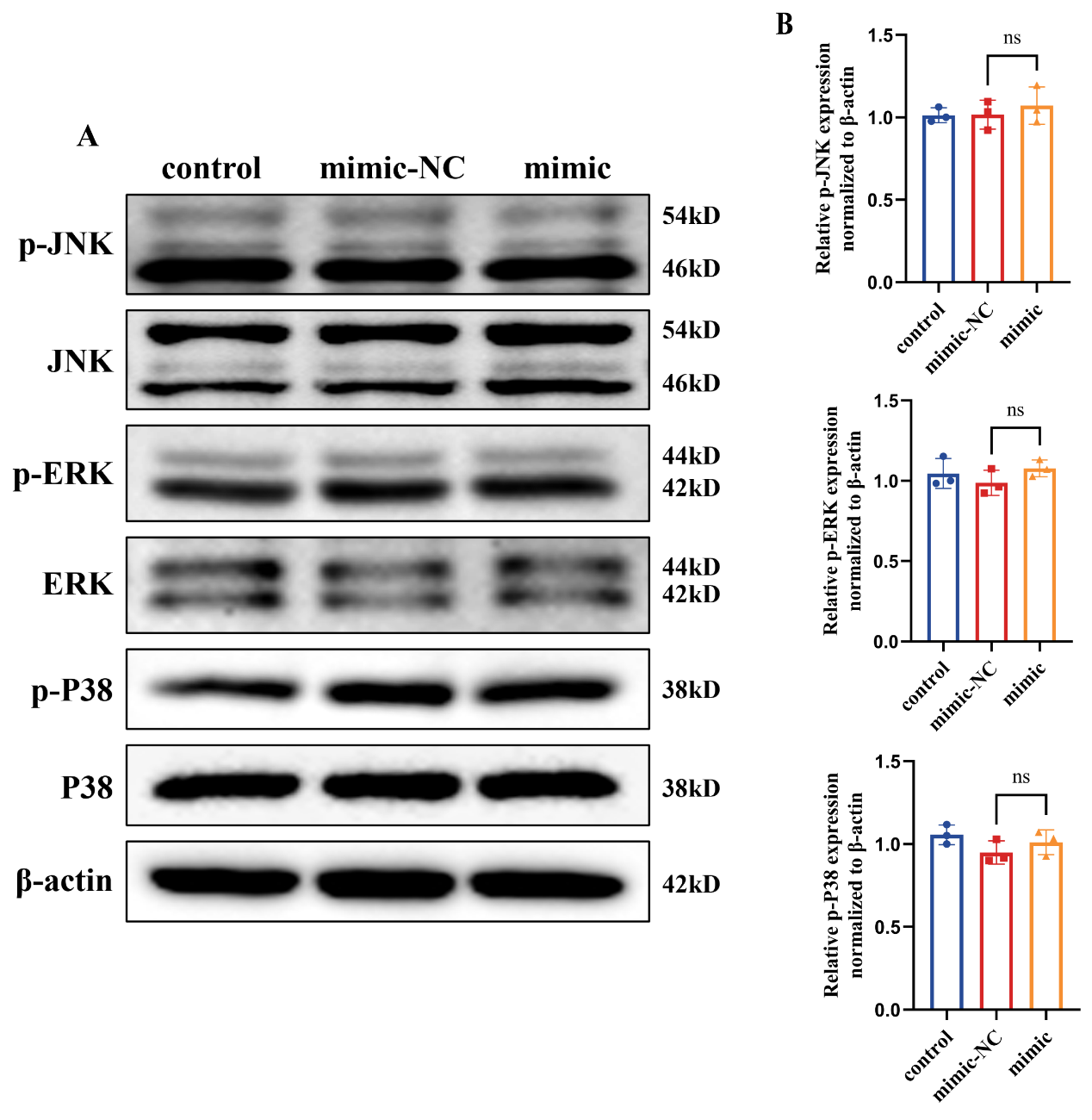
Figure S6: Overexpression of tRF-AspGTC does not activate the MAPK pathway in VSMCs**

(A) WB analysis of MAPK pathway proteins after tRF-AspGTC overexpression. (B) Quantitative analysis of Figure A; n=3 per group.
